# FLUTE: A Python GUI for interactive phasor analysis of FLIM data

**DOI:** 10.1017/S2633903X23000211

**Published:** 2023-11-06

**Authors:** Dale Gottlieb, Bahar Asadipour, Polina Kostina, Thi Phuong Lien Ung, Chiara Stringari

**Affiliations:** Laboratory for Optics and Biosciences, École Polytechnique, CNRS, INSERM, Institut Polytechnique de Paris, 91128 Palaiseau, France

**Keywords:** Data visualization, FLIM, fluorescence lifetime microscopy, phasor analysis, Python

## Abstract

Fluorescence lifetime imaging microscopy (FLIM) is a powerful technique used to probe the local environment of fluorophores. The fit-free phasor approach to FLIM data is increasingly being used due to its ease of interpretation. To date, no open-source graphical user interface (GUI) for phasor analysis of FLIM data is available in Python, thus limiting the widespread use of phasor analysis in biomedical research. Here, we present Fluorescence Lifetime Ultimate Explorer (FLUTE), a Python GUI that is designed to fill this gap. FLUTE simplifies and automates many aspects of the analysis of FLIM data acquired in the time domain, such as calibrating the FLIM data, performing interactive exploration of the phasor plot, displaying phasor plots and FLIM images with different lifetime contrasts simultaneously, and calculating the distance from known molecular species. After applying desired filters and thresholds, the final edited datasets can be exported for further user-specific analysis. FLUTE has been tested using several FLIM datasets including autofluorescence of zebrafish embryos and in vitro cells. In summary, our user-friendly GUI extends the advantages of phasor plotting by making the data visualization and analysis easy and interactive, allows for analysis of large FLIM datasets, and accelerates FLIM analysis for non-specialized labs.

## Impact Statement

This work introduces the first open-source graphical user interface (GUI) for phasor analysis of fluorescence lifetime microscopy (FLIM) data programmed in Python, one of the most widely used programming languages. Phasor analysis is being increasingly used for FLIM data analysis in biomedical research because it reduces the complexity of the analysis and provides a unique visualization of the data with respect to multi-exponential fitting. However, the adoption of FLIM in the life sciences has been hampered by the lack of open-source and user-friendly GUIs. Here, we introduce Fluorescence Lifetime Ultimate Explorer (FLUTE) that simplifies phasor FLIM image processing, accelerates the whole FLIM analysis, and simplifies the visualization and the analysis of FLIM data, thus making phasor analysis possible for a broader base of researchers. FLUTE will be of interest to researchers with interests ranging from physics to biology and will facilitate research in several biomedical fields. Moreover, being open-source and written in Python, FLUTE supports code reusability and the extensibility within the growing biophysics and biophotonics community.

## 1. Introduction

Fluorescence lifetime imaging microscopy (FLIM) is a powerful technique used to probe the local environment of fluorophores reporting on pH, fluorescence resonance energy transfer (FRET)*,* binding, and metabolism.^(^^1^ ^–^^5^ ^)^ FLIM is increasingly used in biology and biophysics applications because the fluorophore lifetime can be used to provide functional contrast in images. However, the use of quantitative, easy, fast, and interactive analysis of large FLIM datasets has been until now hampered by the lack of suitable open-source software.

The most intuitive method to analyze FLIM data is to fit the complex fluorescence decay curve measured in every pixel of the image with a multi-exponential function.^(^^3^ ^,^^5^ ^)^ Several companies have developed commercial closed-source packages for multi-exponential FLIM analysis that usually support their own file formats^(^^6^ ^)^ and recently some free and open-source software options have been developed for exponential fitting such as FLIMfit,^(^^7^ ^)^ FLIM–FRET analyzer,^(^^8^ ^)^ Flimview^(^^9^ ^)^, and FLIMJ.^(^^10^ ^)^

An alternative to multi-exponential curve fitting that has been gaining popularity is the phasor approach^(^^11^ ^–^^17^ ^)^ as it reduces the complexity and calculation time of the analysis while providing a unique visualization option.^(^^5^ ^,^^17^ ^–^^20^ ^)^ The phasor approach is a fit-free technique in which the fluorescence decay from each pixel is transformed with a fast Fourier transform (FFT) into a point in two-dimensional phasor space. The main advantages of phasor analysis are that it provides a visual distribution of the molecular species by clustering pixels with similar decays, it allows for color mapping of the pixels of the FLIM images, and it is linear in terms of non-interacting molecular species.^(^^15^ ^,^^16^ ^,^^21^ ^,^^22^ ^)^ Phasor analysis of FLIM data is increasingly used in several fields of biomedical research^(^^23^ ^–^^40^ ^)^ and is particularly useful for metabolic imaging with the endogenous biomarkers NAD(P)H and FAD to map their complex autofluorescence distributions in live tissues in the context of different physiopathological processes.^(^^5^ ^,^^16^ ^,^^41^ ^–^^63^ ^)^.

The most commonly used software in biomedical research for phasor analysis is SimFCS,^(^^64^ ^)^ developed by Gratton et al.^(^^17^ ^)^ and which is freely distributed. Recently, Schrimpf et al.^(^^65^ ^)^ introduced PAM, an open-source software package written in MATLAB that also includes phasor analysis of FLIM data, whereas Gao et al.^(^^10^ ^)^ integrated phasor transformation in the ImageJ plugin FLIMJ. While companies have been increasingly developing interfaces to analyze FLIM data acquired with their systems, to date, an open-source and easy-to-use software in Python for phasor analysis of FLIM data is not available.

Here, we developed Fluorescence Lifetime Ultimate Explorer (FLUTE) that is designed to fill this gap, and allow simple, quick, and interactive visualization of FLIM data acquired in the time domain. The user-friendly graphical user interface (GUI) accelerates the FLIM analysis pipeline and allows for analysis of large FLIM datasets through bulk processing. FLUTE will be helpful in microscopy labs to perform real-time phasor analysis during experiments while also allowing for post-processing by exporting the data. It is designed to be accessible for researchers of different backgrounds who may not be specialized in FLIM. Moreover, since FLUTE is open-source and programmed in Python, an extension of the included analysis options and integration of existing code in other open-source Python-based platforms are possible and desired.

## 2. Results

### 2.1. GUI development and implementation

**Figure 1.**
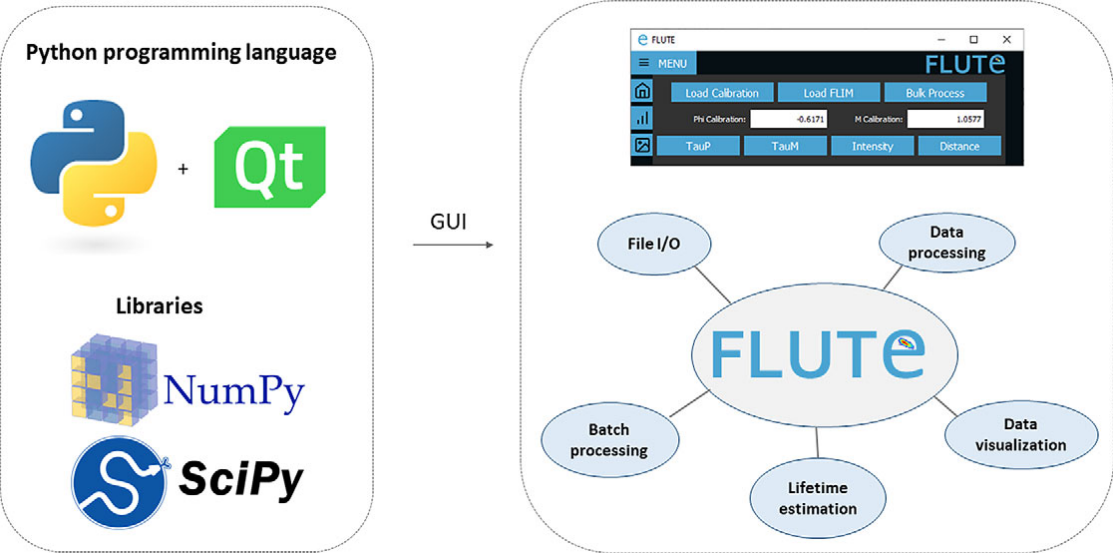
Fluorescence Lifetime Ultimate Explorer (FLUTE) architecture. FLUTE is programmed in Python using PyQt5, NumPy, and SciPy libraries. The graphical user interface is designed to be intuitive and to efficiently perform different functionalities, including data import and export, data processing, data visualization, lifetime estimation, and batch processing. All these functionalities are integrated into a single minimalistic software.

FLUTE is programmed in Python, which offers a significant benefit thanks to extensive support libraries and large user base. This facilitates software “extensibility,” enabling the integration of supplementary functionalities and modules, or allowing further integration in other open-source Python-based image analysis platforms like napari.^(^^66^ ^)^ Furthermore, Python’s syntax is more straightforward compared to other programming languages, rendering it comprehensible and adaptable for researchers with limited programming background. We used PyQt5^(^^67^ ^)^ for the GUI, while phasor analysis (Section 3.1) and data processing are performed using NumPy^(^^68^ ^)^ and SciPy (Figure 1). Both the Python code and the executable file are free, open-source, and available on our GitHub repository^(^^69^ ^)^ (see the Supplementary Material and Supplementary Figures S1 and S2). The easiest way to use FLUTE is to run the available “FLUTE.exe” file, which has been tested to work on Windows 7, 10, and 11 and does not require Python to be installed. The code “main.py” can also be run after installing Python (tested with Python 3.10) and installing all the necessary packages (PyQt5, numpy, opencv-python, matplotlib, and scikit-image). Running “main.py” has been tested to work on different operating systems: Windows, Linux, and MacOS including M1 and M2 chips. A description of an easy procedure to run main.py with a Mac and/or Windows by downloading all the necessary files from FLUTE’s GitHub page, as well as troubleshooting to run main.py with Linux, can be found in the Supplementary Material (see Section 1.2 of the Supplementary Material).

**Figure 2.**
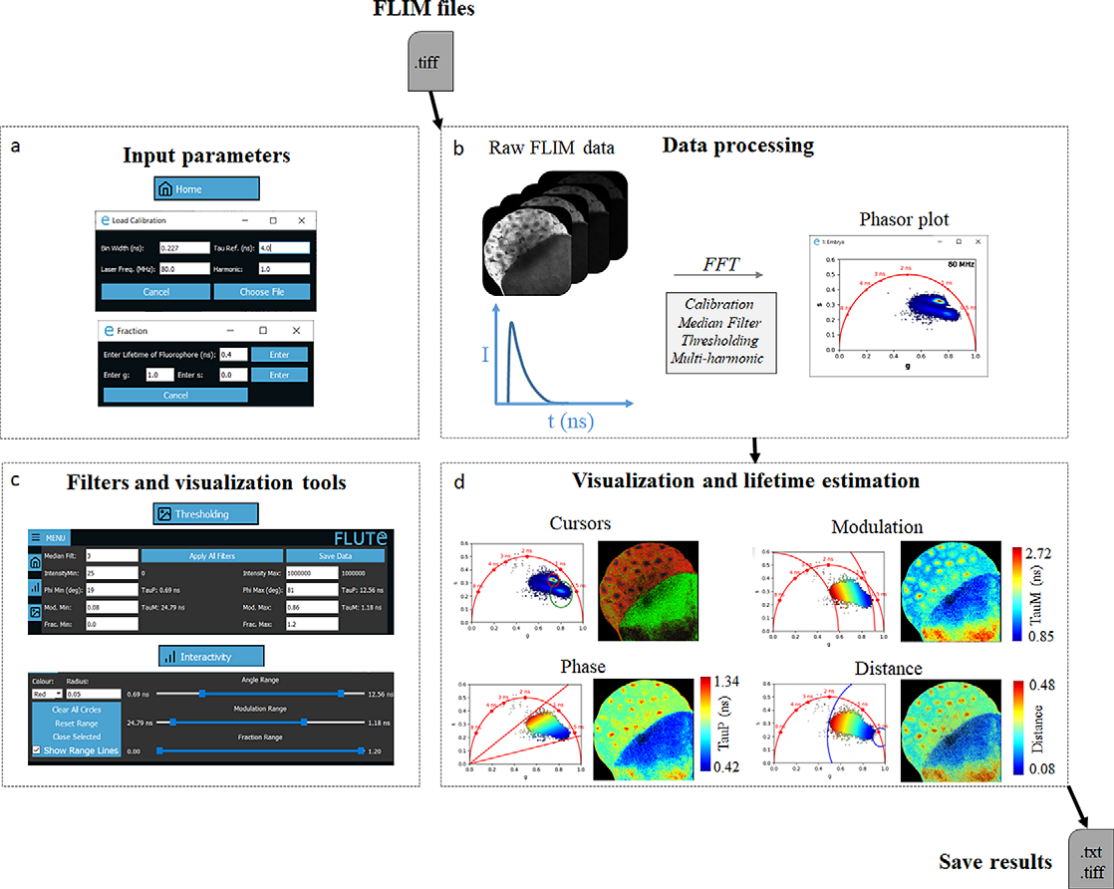
Fluorescence Lifetime Ultimate Explorer (FLUTE) interface for data processing and visualization. FLUTE architecture is purposefully designed and optimized for user-friendly and efficient fluorescence lifetime imaging microscopy (FLIM) data processing and visualization. (a) Window of FLUTE interface that allows choosing input parameters to calibrate and calculate the distance from known molecular species. (b) Data processing enables the import of raw FLIM data in .tiff format, performs phasor transformation with a fast Fourier transform at different harmonics of the laser repetition frequency, and applies reversible median filter and intensity thresholding. (c,d) FLUTE offers a range of tools for interactive, straightforward, and reproducible data visualization and lifetime estimation such as cluster analysis with cursors of adjustable size and applying various colormaps (distance from molecular species and lifetime contrast TauP and TauM) interactively using range sliders or thresholding entry boxes. These FLIM data were calibrated with fluorescein (4 ns) acquired in solution: Fluorescein_Embryo.tiff.

FLUTE performs phasor analysis on FLIM data in the time domain, either acquired with a time-correlated single photon counting (TCSPC) electronic cards or with the time-gating technique, provided that an entire period of the laser repetition is recorded and the lifetime decay it is not truncated. FLIM data are read as a .tiff stack format (Figure 2b), where each image of the stack represents a temporal bin of the FLIM stack acquired in the time domain. FLIM data acquired with commercial cards that are not already in a .tiff format have to be first converted using either the associated commercial software or available open-source plugins (see Section 11 of the Supplementary Material).

The FLUTE architecture has been designed to perform several tasks and processes in a user-friendly way (Figure 1). New and key features of FLUTE, in comparison to other software tools for phasor analysis, are highlighted in bold below and in Table 1 in Section 12 of the Supplementary Material.

*File I/O*

- Reading FLIM files (.tiff)
- Exporting results (analysis and figures)

*Data processing*

- Calibration (with a reference standard)
- Phasor transformation at different harmonics
- Filters (median filter, thresholds, etc.) applied in a reversible way

*Data visualization*

- Interactive exploration of data with cluster analysis with cursors of adjustable size
- **Simultaneous display of phasor plot and FLIM images with different lifetime contrast**
- **Interactive change of the contrast with thresholding entry boxes or range sliders**

*Lifetime estimation*

- **Calculation of tauPhase (TauP) and tauModulation (TauM) lifetimes**
- **Reproducible calculation of distance from known molecular species**
- Calculation of average lifetime in ROIs

*Batch processing*

- Processing and analyzing large FLIM datasets.

FLUTE introduces novel tools and features that distinguish it from previous software. These additions enable interactive, straightforward, and reproducible FLIM analysis. In particular, we introduced (a) an interactive visualization of lifetime displays TauP and TauM (Figure 2d); (b) a precise mapping of the distance from known molecular species by defining their location in the phasor plot, allowing to calculate the fraction of molecular species; and (c) interactive simultaneous display of the color map of phasor plot and FLIM images using thresholding entry boxes or range sliders (Figure 2c,d).

### 2.2. FLIM data processing

FLUTE has been programmed to perform phasor analysis of FLIM data (Section 3.1) acquired in the time domain through an FFT (Figure 2a,b). Phasor coordinates *g* and *s* are calculated by using (1) and (2) (Section 3.1). The main window of FLUTE is shown in Figure 1 and Supplementary Figure S3. First, FLIM data need to be calibrated using a fluorescence calibration standard of known lifetime to take in account microscope temporal response, which includes the response of the detectors and the TCSPC electronics^(^^17^ ^)^ (see Section 3). The calibration can be performed through the measurements of the impulse response function with a second-harmonic generation (SHG) signal by using samples such as KDP, urea crystals, or starch. As SHG is an instantaneous process, its lifetime is considered 0 ns. Alternatively, the calibration can be performed with fluorophores with a single-exponential decay. A compilation of fluorescence lifetime standards ranging from a few hundreds of picoseconds to a few nanoseconds can be found in the literature.^(^^70^ ^–^^72^ ^)^ Typical single-exponential fluorophores that are used for calibration are Fluorescein (4 ns), Coumarin 6 (2.5 ns), Rhodamine B (1.74 ns), or Rose Bengal (0.52 ns). The choice of the fluorophore for calibration is mainly determined by the excitation wavelength used in the experiments, and the range of lifetime that needs to be measured. To perform the most accurate calibration, the reference lifetime should be chosen in the same range of the sample lifetime that are going to be measured; for example, to measure accurately the lifetime shorter than 1 ns, a calibration reference with SHG signal (0 ns) is preferable. Moreover, as the lifetime is affected by parameters such as temperature, buffer, pH, and concentration, careful calibration is important to assure a correct referencing and to avoid biases generated experimentally.

The FLIM data are converted to the phasor plot with an FFT and referenced using the fluorescence calibration standard of known single lifetime (Figure 2a,b and Supplementary Figure S4). We note that it is crucial to acquire the FLIM image with the same experimental parameters that were used for calibration (i.e., laser excitation wavelength, phase settings in the TCSPC software, as well as temporal bin number and bin width), as using different parameters would lead to an incorrect absolute position of the FLIM data in the phasor plot. FLUTE allows for analysis of FLIM data acquired with different parameters such as temporal bin number, bin width, laser repetition rates (in megahertz), and different harmonics of the FFT transformation. These parameters need to be specified in the GUI in the “Load Calibration” (Figure 2a and Supplementary Figure S4) during the calibration step. The bin number and laser repetition rate parameters can usually be found in the metadata of the FLIM files (see Section 11 of the Supplementary Material), while the bin width should be calculated in nanoseconds using the following formula: bin width = 1/(laser repetition rate × bin number).

The default harmonic of the FFT calculation is set at 1, therefore performing the calculations with the same frequency of the laser repetition rate. FLUTE displays the FFT frequency used in the top corner of the phasor plot (see Figure 2b). We implemented the option to perform Phasor analysis of the FLIM data at higher harmonics of the laser repetition rate by changing the “harmonic” parameter in the calibration window of FLUTE (Supplementary Figures S4 and S5) during the calibration step. Higher harmonic phasor analysis can be useful to separate and identify multi-exponential tissue component present in complex tissues.^(^^16^ ^)^ An example of FFT transformation with higher harmonics is represented in Supplementary Figure S5b.

When FLIM data are first opened and processed with the phasor transformation (Supplementary Figure S6), the initial phasor plot is displayed and color mapped as a histogram showing the density of points in phasor location and the initial intensity image is calculated as the sum of the .tiff stack (Supplementary Figure S6). Processing times with a MacBook Air (M1, 2020, 8 GB Memory) for a phasor transformation of FLIM stacks are ~0.7 s for a 256 × 256 image, ~1.1 s for a 512 × 512 image, and ~3 s for a 1,024 × 1,024 image with 56 time beans each (see Section 13 of the Supplementary Material).

FLUTE can also apply a threshold intensity filter (Supplementary Figure S9) and a reversible 3 × 3 convolutional median filter on the phasor *g* and *s* coordinates (Supplementary Figure S8) to reduce the spread of phasor points and reduce noise without affecting the resolution of the image.^(^^17^ ^,^^21^ ^)^

### 2.3. FLIM data visualization and lifetime estimation

We designed FLUTE to be an intuitive GUI for flexible data exploration and easy data visualization (Figure 2c,d). We introduced novel tools and features that enable interactive, straightforward, and reproducible FLIM analysis. Figure 2d illustrates an example of FLIM data visualization using FLIM data from the autofluorescence of a Zebrafish embryo at 3 h post-fertilization (3hpf), that is uploaded on our Zenodo repository (https://zenodo.org/record/8324901) as *Embryo.tiff* stack. This example highlights the capabilities of FLUTE software to explore complex multi-exponential FLIM data without a priori knowledge on the tissue content and on the type of fluorophores present in tissues.

Figure 2c also shows the corresponding FLUTE user interface that is used to perform this analysis. Interactive exploration of the FLIM data can be performed by using multiple colored cursors of variable sizes to select pixels with similar fluorescence decays and phasor locations, highlighting simultaneously corresponding pixels in the image by reciprocity principle (Figure 2, Supplementary Figure S17, and Section 8.4 of the Supplementary Material).

FLUTE also allows to estimate the lifetime of the FLIM image and to choose the appropriate color mapping of phasor plot and FLIM images for data exploration (Supplementary Figures S3 and S10). TauP is estimated with (9) and TauM with (10), respectively. The threshold of these color maps for TauP and TauM can be adjusted either via the Interactivity window (Figure 2c and Supplementary Figure S11) or via the Thresholding window (Figure 2c and Supplementary Figure S12) of the GUI. Mapping of the FLIM image and of the relative phasor plot according to lifetime contrasts such as TauP or TauM is performed simultaneously by using thresholding entry boxes or range sliders (Figure 2c), allowing for a clear representation and an interactive exploration of the FLIM data (Sections 8.1 and 8.2 of the Supplementary Material).

**Figure 3.**
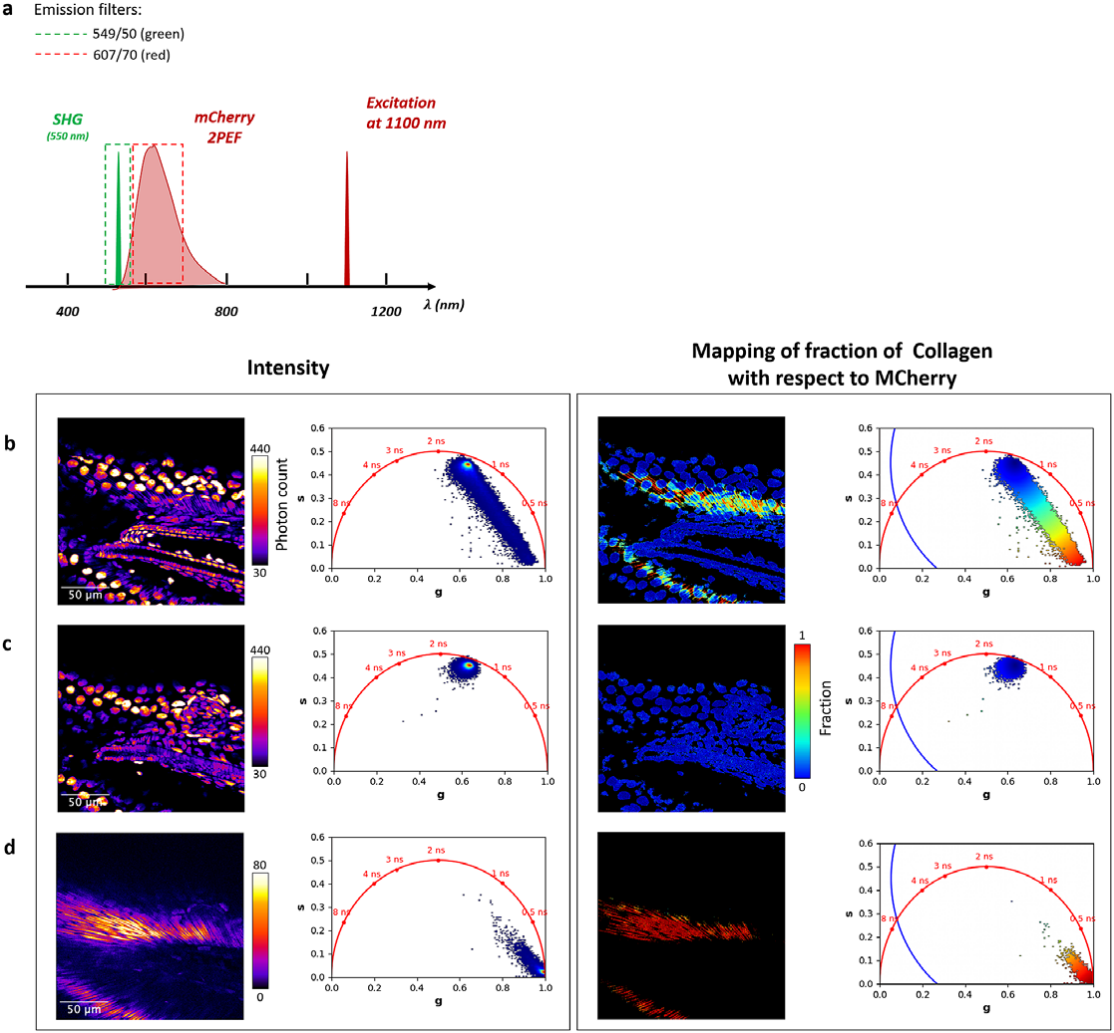
Mapping of distance from mCherry in a 5 days post-fertilization zebrafish embryo tail (H2B-mCherry line). (a) Excitation and emission scheme of the experiment. Two-photon excitation of mCherry and second-harmonic generation (SHG) of collagen is performed at 1,100-nm wavelength. Images are acquired without emission filter to collect both mCherry from the cell nuclei and SHG from collagen (b), with an emission filter of 607/70 nm to collect only mCherry fluorescence (c) and with an emission filter of 549/50 nm to collect only the SHG signal from collagen. (b–d) Intensity images and distance from mCherry with their corresponding phasor plots are displayed for the images with mCherry and collagen (b), mCherry only (c), and collagen only (d). These fluorescence lifetime imaging microscopy images were calibrated with the SHG signal (0 ns) acquired from a starch sample: starch SHG-IRF.tif. The distance 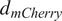 from mCherry is calculated with Fluorescence Lifetime Ultimate Explorer using equation (15) from the mCherry phasor location (g = 0.634 and s = 0.45) estimated from the average phasor plot of (c).

A mixture of two fluorescent non-interacting molecular species (i.e., excluding FRET) gives rise to a linear combination in the phasor space (see Section 3.1). FLUTE can calculate and map the distance 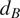 from the molecular species (B) by using (15) and by either inserting its single lifetime or its phasor coordinates (Figure 9, Supplementary Figure S15, and Section 8.3 of the Supplementary Material) with a single or a multi-exponential decay, respectively. If the users want to calculate the fraction 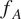 of molecular species A with respect to B, the distance 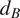 from molecular species B calculated by FLUTE needs to be manually normalized by the distance between species A and B (see Section 3.1) by using (16). An example of quantification of the distance from mCherry in a live Zebrafish embryo at 5 days post-fertilization (5dpf) from an H2B-mCherry line (see Section 3.2) is represented in Figure 3. The tail of the H2B-mCherry zebrafish line is excited at 1,100 nm, and FLIM images are collected with different emission filters to selectively collect mCherry from cell nuclei and/or SHG from collagen. *ZF-1100_noEF.tif, ZF-1100_607-70_filter.tif*, and *ZF-1100_550-49_filter.tif* that correspond to Figures 2b–d, respectively, are uploaded in our Zenodo repository (https://zenodo.org/record/8324901). It is possible to manually calculate and map the fraction of collagen 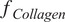 with respect to mCherry by normalizing the distance from mCherry 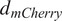 by the distance between mCherry and collagen. By estimating the phasor location of SHG from Collagen from *Figure 3d*, *g* = 1 and *s* = 0, which corresponds to 0 ns lifetime, the distance between mCherry and collagen is calculated to be 0.58.

Figures 5 in section 2.5 we illustrate examples of data visualization and mapping of the endogenous metabolic coenzyme NADH in cell culture, which is an in vitro assay often used to perform metabolic imaging. The distance 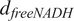 from free NADH can be calculated and mapped graphically in every pixel (Figures 2, 5 section 2.5 and Supplementary Figure S16) from the location of the free NADH that has a known single lifetime of 0.4 ns^(^^73^ ^)^ (see Section 3.1). It is possible to manually calculate the fraction of bound NADH 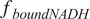 as the ratio between the distance 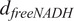 from free NADH and the distance between the phasor location of bound NADH and the location of the phasor location of free NADH. However, while the lifetime of free NADH in solution is considered stable and used as a reference, the lifetime of bound NADH is complex and highly dependent on enzymatic binding, cell type, and differentiation state.^(^^3^ ^,^^5^ ^,^^6^ ^,^^73^ ^,^^74^ ^)^ Therefore, the estimation of fraction of bound NADH 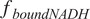 can be performed by a two-component analysis only when the location of bound NADH is assumed.^(^^17^ ^,^^45^ ^,^^51^ ^,^^75^ ^–^^77^ ^)^ As the precise location of bound NADH is not known, it is often preferable to simply estimate the distance 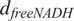 from the location of free NADH (Section 2.5).^(^^41^ ^,^^42^ ^,^^61^ ^,^^78^ ^)^ We note that 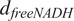 and 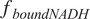 represent relative degrees of enzymatic binding, but they are not dependent on the overall concentration of NADH.

### 2.4. Saving results and batch processing

**Figure 4.**
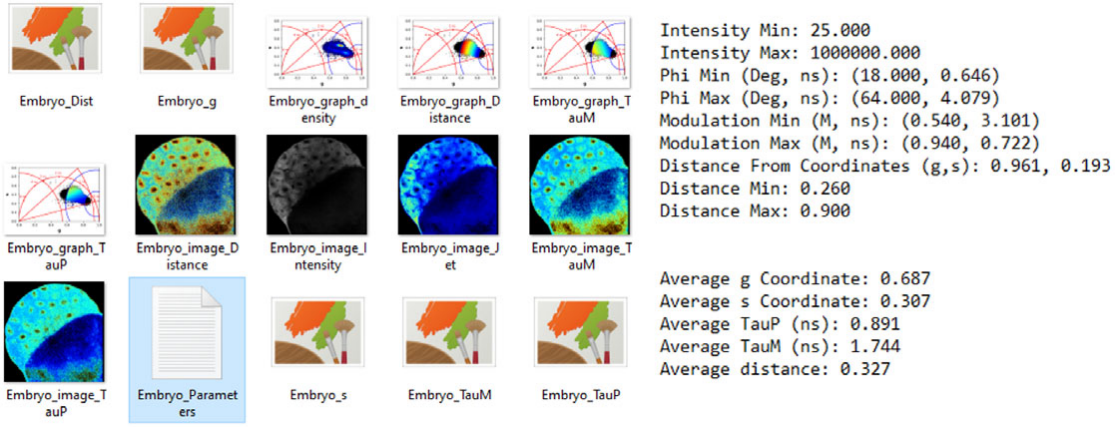
Fluorescence Lifetime Ultimate Explorer result export. Saved fluorescence lifetime imaging microscopy images and phasor plots (left) and applied filters (right) to create the mask and measurements of the average of g, s, TauP, TauM, and distance (right).

We implemented image masking by applying thresholds on the lifetime values or on the distance from molecular species. Once the desired thresholding is applied on the FLIM image (Supplementary Figure S18), the data with the applied mask can be saved (Figures 2 and 4 and Section 9.1 of the Supplementary Material). FLUTE saves the matrices of phasor coordinates, the lifetime contrasts, and the distance of the molecular species in .tiff files as well as the calculated corresponding average values in the mask (Figure 4 and Section 9.3 of the Supplementary Material). Finally, FLUTE allows batch processing on multiple FLIM images analyzed using the same parameters (Supplementary Figure S21 and Section 10 of the Supplementary Material). Typical processing times for the full analysis of one image (that includes phasor transformation, applications of filters, saving results, images, and measurements) are ~1.8 s for a 256 × 256 image, ~2.5 s for a 512 × 512 image, and ~5.4 s for a 1,024 × 1,024 image with a MacBook Air (M1, 2020, 8 GB Memory) (see Section 13 of the Supplementary Material).

### 2.5. FLIM analysis of NADH autofluorescence in live cells

**Figure 5.**
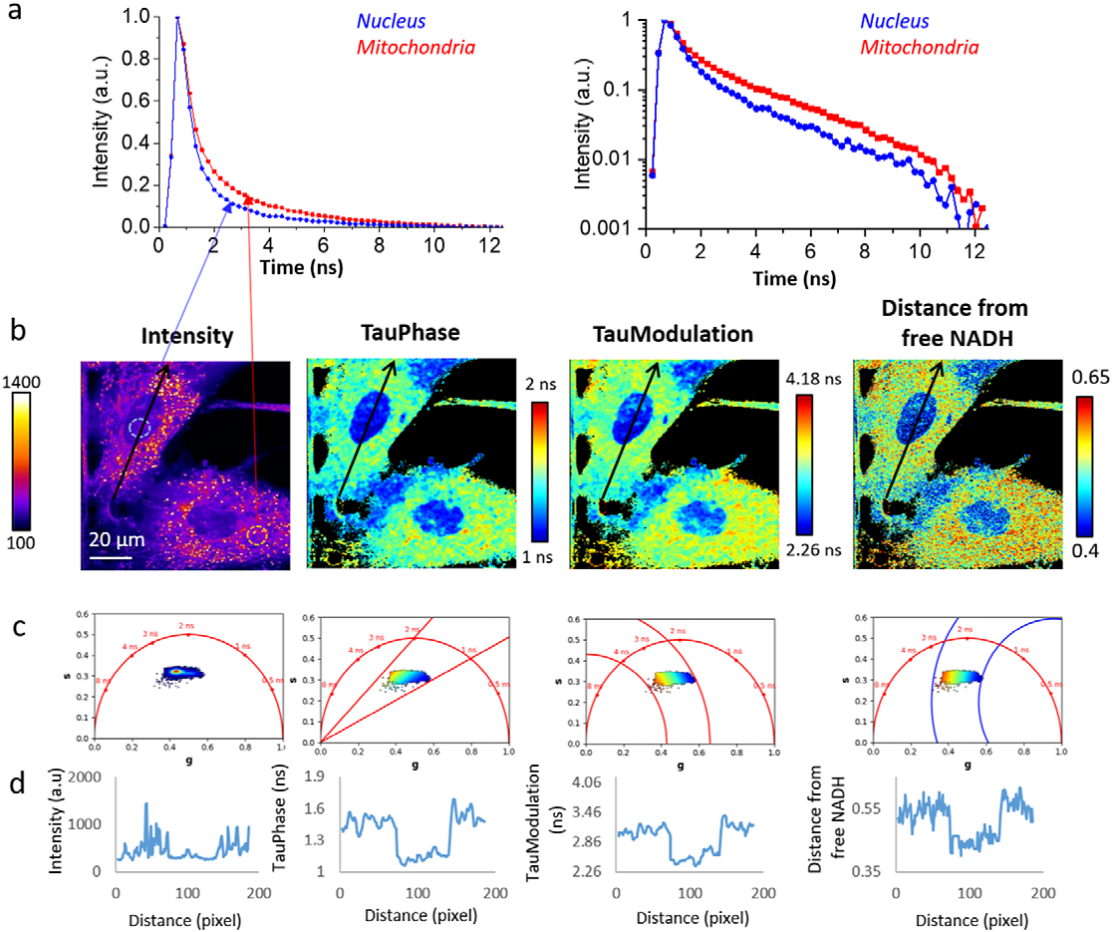
Fluorescence lifetime imaging microscopy (FLIM) analysis of NADH reveals intracellular metabolic heterogeneity in mesenchymal stromal cells. (a) Multi-exponential fluorescence intensity decays of the intrinsic biomarker NADH in the nucleus and in the mitochondria of mesenchymal stromal cells. Linear scale (left) and logarithmic scale (right). (b,c) Cellular maps (b) and corresponding phasor plots (c) are displayed with different contrasts: intensity, TauP lifetime, TauM lifetime, and distance from free NADH after applying an intensity threshold of 200 and 3 median filters. This FLIM image was calibrated with fluorescein (4 ns) acquired in solution: Fluorescein_hMSC.tif. (d) Profiles of the respective contrast along the black line drown in (b).

**Figure 6.**
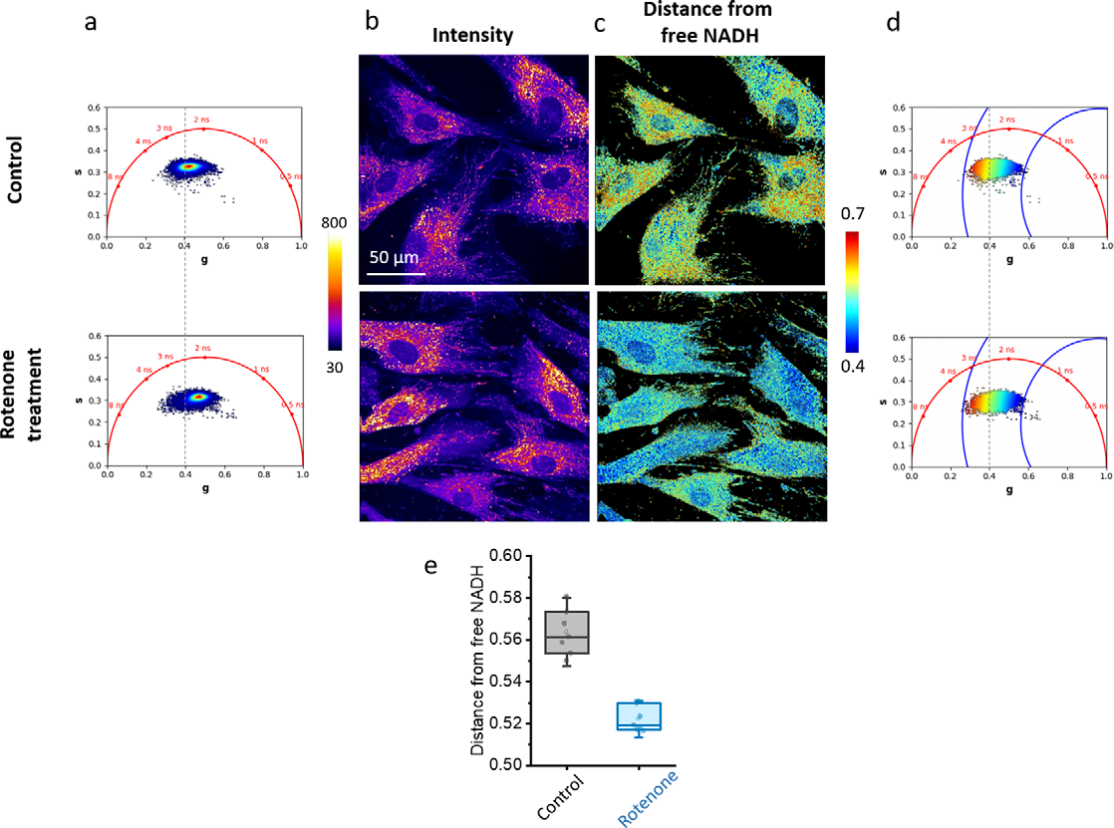
Shift in the distance from free NADH in live cells upon metabolic treatment. (a,b) Phasor plot (a) and the corresponding intensity images (b) of hMSCs with different treatments: control and rotenone (respiratory chain inhibitor). (b,c) Images (c) and the corresponding phasor plots (d) are mapped with the distance from free NADH contrast after applying an intensity threshold of 100 and 3 median filters. These fluorescence lifetime imaging microscopy images were calibrated with fluorescein (4 ns) acquired in solution: Fluorescein_hMSC.tif. (e) Measurement of the average value of the distance from free NADH in seven ROIs for each condition. T-test is performed (**P < .01).

We tested FLUTE on a FLIM dataset acquired in human mesenchymal stromal cells (hMSCs) in vitro^(^^61^ ^)^ to demonstrate the ability of the software to visualize the autofluorescence of the intrinsic biomarker NADH^(^^73^ ^,^^79^ ^)^ and quantify metabolic states associated with subcellular compartments (Figure 5) and metabolic shifts associated with drug treatments (Figure 6).

Figure 5 illustrates an example using FLIM data from hMSCs that is uploaded on our Zenodo repository (https://zenodo.org/record/8324901) as *hMSC* *-ZOOM* *.tif* stack. We measured multi-exponential fluorescence intensity decays of NADH with a shorter lifetime in the nucleus with respect to the mitochondria (Figure 5a). Using FLUTE, we estimated and visualized the NADH lifetime and the relative fraction of bound/free NADH in hMSCs by mapping both the images (Figure 5b) and the corresponding phasor plot (Figure 5c) with TauP ( 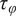 ), TauM ( 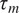 ) lifetimes, and the distance from free NADH contrasts calculated with (9), (10), and (15), respectively. This analysis reveals subcellular metabolic heterogeneity on a pixel level and a shorter NADH lifetime and a smaller distance from free NADH in the nucleus with respect to the mitochondria (Figure 5b–d), as previously observed in the literature.^(^^80^ ^)^ Maps of NADH lifetime and distance from free NADH can be exported with FLUTE and used for further image processing. For example, we measured the profiles of the abovementioned parameters along a line across mitochondria and nucleus (Figure 5b,d). The values of 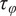 vary from 1 ns (in the nucleus) and 1.3–1.7 ns in the mitochondria, the values of TauM, 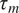 , vary from 2.2 to 3.4 ns in the mitochondria, whereas the distance from free NADH vary from 0.45 (in the nucleus) to 0.55 (in the mitochondria).

Figure 6 illustrates an example using FLIM data from the NADH autofluorescence of mesenchymal stromal cells treated with the metabolic drug rotenone, and the data are uploaded on our Zenodo repository^(^^81^ ^)^ as *hMSC_control.tif and* hMSC*_rotenone.tif* stacks. We estimated the metabolic ratio of bound/free NADH by mapping at the pixel level the distance from free NADH (Figure 6c,d) calculated from the phasor location of free NADH (Figure 9b and (15)). Blocking the respiration chain with rotenone leads to a shift of the NADH phasor plot toward the free NADH location (0.4 ns), decreasing the distance from free NADH in the cells (Figure 6c,d). We then performed the batch analysis of several ROIs in control condition and rotenone treatment. With then measured the average value of the distance from free NADH in every ROI, and we performed a statistical analysis in Figure 6e, showing that the two conditions are significantly different.

## 3. Methods

### 3.1. Phasor analysis

**Figure 7.**
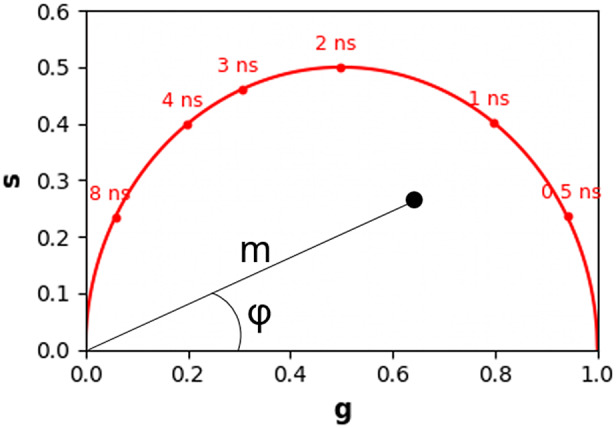
Phasor plot representation. Single-exponential decays fall on the semicircle (red points), while multi-exponential decays are located within the universal circle of the phasor plot (black point). The phasor coordinates g and s are the real and imaginary parts of the fast Fourier transform.

The multi-exponential intensity decay of every pixel of the FLIM image acquired in the time domain is converted in the phasor plot with a Fourier transform using the following formulas:(1)

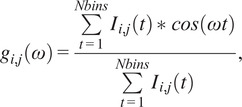


(2)

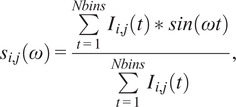

where *i* and *j* indicate the order pixel of the image, *ω* is the frequency of the FFT calculation, and Nbins is the total number of time bins. *ω* is calculated through the following expression: *ω* = 2π*n* *f*, where *f* is the laser repetition rate and *n* is the number of the harmonic. *T* is the period of excitation: *T* = 1/*f.* The real (*g*) and imaginary (*s*) parts are plotted in the graphical phasor plot (Figure 7). In the frequency domain, the FLIM data in the frequency domain can be expressed as follows:(3)

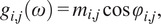


(4)

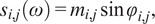

where the *φ_i,j_* and *m*_*i*_ are the phase and the modulation of the emission with respect to the excitation.

In the case of a single-exponential decay 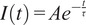 , the coordinates of the phasor are given by the following equations:(5)

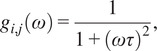


(6)

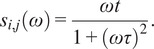

All single-exponential decays fall on the semicircle of radius ½ and center (½,0), commonly called the universal circle (Figure 7).^(^^15^ ^)^ A short lifetime having a smaller phase *φ* will lie next the point (1,0), which corresponds to *τ* = 0, while a long lifetime will fall near the universal circle coordinates (0,0), which corresponds to *τ* = ∞.^(^^15^ ^)^ The phasor location of a multi-exponential lifetime will fall inside the universal circle as represented by the black point in Figure 7.

The phase and the modulation of a decay in the phasor plot are calculated using the following equations:(7)

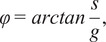


(8)

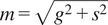

Estimations of the lifetime in each pixel in terms of the phase and the modulation can be performed by(9)

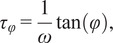


(10)

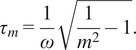

**Figure 8.**
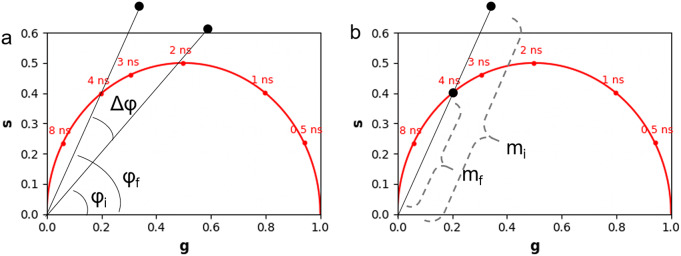
Calibration of the phasor plot using a fluorescent standard of fluorescein of 4 ns. (a) Phase correction. (b) Modulation correction.

The phase ( 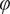 ) and the modulation (*m*) of the phasor cloud are first referenced using calibration to account for the instrument response function and the delays of the electronics. The calibration is performed with a reference sample that is usually a fluorophore with a known fluorescence lifetime, for example, fluorescein that has a decay of 4 ns or with an instrument response function like the SHG measurement of 0 ns. Two examples of fluorescein decay are uploaded on our Zenodo repository^(^^81^ ^)^ as *Fluorescein_embryo.tif* and *Fluorescein_hMSCs.tif*; they are used to calibrate the FLIM data in the embryo and in hMSCs, respectively. Figure 8 demonstrates the principle of the phasor calibration with Fluorescein: a correction on the phase is performed applying an offset of 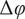 , while correction on the modulation is performed applying a multiplication constant 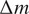 as described in the following formulas:(11)

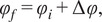


(12)

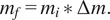

**Figure 9.**
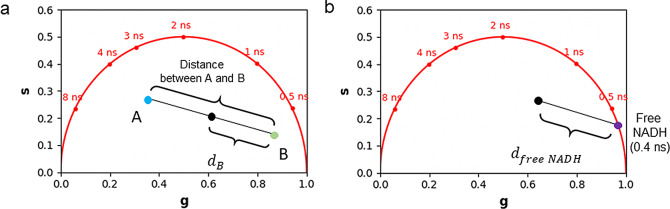
*Graphical calculation of the distance from a molecular species. (a) The distance from molecular species B (* 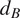 *) is defined as the distance of the experimental point (black point) from molecular species B. The fraction* 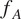 *of molecular species A is defined as the distance dB normalized by the distance between points A and B. (b) graphical calculation of the distance* 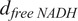 *from free NADH.*

If the FLIM image contains a combination of two non-interacting fluorescent molecular species (i.e., excluding FRET), it gives rise to a linear combination in the phasor space (Figure 9). In a system with two fluorescent species A and B, the phasor of the experimental point (black point) lies along a straight line joining the phasors of the two species A and B.(13)

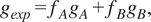


(14)

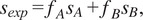

where 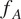 is the fractional contribution of molecular speaie A and 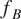 is the fractional contribution of molecular species B.

The distance from molecular species B ( 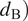 is graphically calculated as the distance between the black experimental point ( 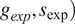 and the location of the molecular species B ( 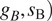 using the following equation:(15)

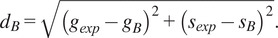

The fraction of molecular species A ( 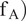 is defined as the distance 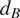 normalized by the distance between point A ( 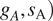 and point B ( 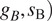 and calculated using the following formula:(16)

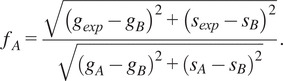

We note that the above calculated distance and fraction are not weighed by the quantum yield of the molecular species.

### 3.2. Two-photon excited fluorescence lifetime microscopy imaging of zebrafish embryo and mesenchymal stromal cells

Fluorescence lifetime microscopy imaging was performed with a laser scanning microscope (TriMScope, Lavision Biotec, Bielefeld, Germany) equipped with a dual-output femtosecond laser (Insight DS++, Spectra-Physics, Santa Clara, CA, USA) with a tunable beam from 680 to 1,300 nm (120-fs pulses, 80 MHz) and a fixed beam at 1,040 nm (200-fs pulses). The excitation laser is focused on the sample through a water immersion objective (25X, NA = 1.05, XLPLN-MP, Olympus, Japan). The fluorescence signal is collected through the same objective and then epi-detected by a hybrid photomultiplier tube (R10467U, Hamamatsu, Japan). FLIM is performed with TCSPC electronics (Lavision Biotec). The laser trigger is taken from the fixed wavelength beam using a photodiode (PDA10CF-EC, Thorlabs, Newton, NY, USA). We used a fluorescein solution at pH 9 with a single-exponential of 4.04 ns excited at 740 or 760 nm or SHG signal from a starch sample with a lifetime of 0 ns excited at 1,100 nm to measure a reference lifetime and calibrate the FLIM system. We performed FLIM imaging of a wild type line (AB strain) at 3hpf with 740-nm excitation wavelength with a typical power of 25 mW and collected the fluorescence signal through a band pass filter Semrock FF01-460/80. We performed FLIM imaging of a zebrafish line Tg(ubb: H2B-mCherry) at 5dpf^(^^82^ ^)^ with 1,100-nm excitation wavelength with a typical power of 8 mW and collected images with different emission filters to selectively collect mCherry and/or SHG from collagen. Images are acquired without emission filter to collect both mCherry and collagen, with an emission filter of 607/70 nm to collect only mCherry fluorescence and with an emission filter of 549/50 nm to collect only the SHG signal from collagen. We typically collected 400–800 photons during an acquisition time of 80 s for a 512 × 512 pixel image with a pixel dwell time of 162 μs/pixel).

The wild type embryo at 3hpf were kept inside their chorions. Imaging of 3hpf embryos was performed by embedding them in 1.5–2% Low Melting Point agarose. H2B-mCherry zebrafish embryos were treated with 1-phenyl 2-thiourea 0.003% (P7629, Sigma-Aldrich, St. Louis, MO, USA) in husbandry water when they reached 9 h post-fertilization to inhibit melanogenesis.^(^^83^ ^)^ Before each imaging experiment at 5dpf, the embryos were anaesthetized in a tricaine solution (0.016% in husbandry water) (MS-222 Sigma, St. Louis, MO, USA) in 15–30 min. Imaging of 5dpf embryos was performed by embedding them in 1.5–2% Low Melting Point agarose and adding tricaine (MS-222 Sigma, cat E10521, St. Louis, MO, USA) to a final concentration of 0.02%.

We performed FLIM imaging of human mesenchymal stromal cells (hMSCs) autofluorescence with 760-nm excitation wavelength with a typical power of 12 mW and collected the fluorescence signal through a band pass filter Semrock FF01-460/80. We typically collected 500 photons for FLIM images of hMSCs with a pixel dwell time of 240 μs and a total acquisition time of the order of 1 min.^(^^61^ ^)^ hMSCs were maintained in LG-DMEM (cat. no. 31600-034; Gibco, Waltham, MA, USA) supplemented with 10% FBS, 4-mM L-glutamine, 100 μg/ml of penicillin and streptomycin (Gibco) and incubated at 37°C and 5% of CO2. During the imaging experiments, we used a basal medium without the phenol red. To perform metabolic perturbation, MSCs were treated with rotenone 50 μM in DMSO (R8875, Sigma-Aldrich) to block the respiratory chain through complex I.

## 4. Conclusion

In this article, we presented FLUTE, a highly interactive GUI that removes barriers in FLIM analysis and makes the phasor approach more accessible for applications in biology and the biomedical field. FLUTE performs phasor data analysis acquired in the time domain, both acquired by TCSPC and time-gaiting, provided that the fluorescence decay is properly acquired over the entire period of the laser repetition rate. As FLUTE is open-source and written in Python, it allows other programmers to extend the FLUTE code with complementary advanced developments, introduce an online version with Jupiter notebook, and integrate phasor analysis in other Python open-source image analysis platforms such as napari.^(^^66^ ^)^

FLUTE efficiently provides phasor FLIM data processing and visualization tools (Figure 2) such as calibrating the FLIM data, displaying the phasor plot, calculating the distance from known molecular species and the fraction of molecular species, evaluating lifetime, displaying the phasor plot clouds and the FLIM images with different lifetime contrast simultaneously, performing interactive reciprocity analysis with cursors, applying different types of filters and thresholding, and calculating average lifetime values in masks. In FLUTE, all these functionalities are gathered in the same minimalistic software, allowing for efficient FLIM data analysis without overwhelming the user with other data analysis modules. The main novelty and improvement of FLUTE with respect to previous free and/or open-source software for phasor analysis is the introduction of new metrics, tools, and features to perform interactive, simple, and reproducible FLIM analysis (Table 1 in Section 12 of the Supplementary Material). Among the new tools introduced there are interactive visualization of lifetime displays TauP and TauM, mapping of distance from molecular species by defining the location of molecular species in the phasor plot and highly interactive simultaneous display, and change of the color map of phasor plot and FLIM images with thresholding entry boxes or range sliders (Figure 2b,d). The final edited datasets after applying the desired filters and thresholds can be exported for further user-specific analysis (Figure 4). FLUTE enables simultaneous analysis of multiple images, and it has also been designed to perform batch processing of large FLIM datasets.

We demonstrated that FLUTE is a useful GUI to explore complex multi-exponential FLIM data such as the autofluorescence in a 3hpf zebrafish embryo (Figure 2d) without a priori knowledge on the tissue content and on the type of fluorophores present. We also demonstrated the use of FLUTE to map and quantify the distance from mCherry in the tail of a 5dpf zebrafish embryo (Figure 3) and for metabolic imaging in cells culture to map and quantify the lifetime of NADH and the distance from free NADH at the subcellular level and with drug treatment (Figures 5 and 6).

Overall, FLUTE expands some possibilities of phasor FLIM image processing, accelerates the whole FLIM analysis pipeline, and simplifies the visualization and the analysis of FLIM data, thus making phasor analysis possible for a broader base of researchers.

We strive to keep developing FLUTE software in the future by improving the overall compactness and processing speed, as well as by introducing new modules for advanced analysis. First, in the future version of the FLUTE code, we aim to integrate the direct import of the common FLIM file formats (.std, .fbd, and .ptu) inside the Python code by using already available Python libraries and open-source codes^(^^84^ ^–^^87^ ^)^ (Section 11 of the Supplementary Material) and to introduce an intermediary file format which encompasses matrices for Intensity, *g* and *s* coordinates. In terms of speeds, FLUTE processes and analyzes FLIM data in the order of seconds (~1.1 and ~2.5 s for a phasor transformation and analysis in the bulk process of a 512 × 512 pixels FLIM image). FLUTE phasor processing time represents a significant increase with respect to multi-exponential fitting analysis^(^^19^ ^)^ and performs similarly (in the order of seconds per image) with respect to the open-source phasor software based on central processing units.^(^^10^ ^,^^17^ ^,^^65^ ^)^ It will be possible to increase FLUTE speed with parallel computing capabilities of graphics processing units by using specialized Python libraries such as Numba^(^^88^ ^)^ and CuPy.^(^^89^ ^)^ Further accelerating the processing times to the milliseconds range will allow real-time phasor analysis and representation during experiments.^(^^90^ ^)^ Finally, we aim to adapt the phasor analysis to typical time-gated sampling limitations by taking into account the effect of decay truncation and gate shape,^(^^91^ ^–^^93^ ^)^ integrate a fully automated calculation and mapping of fraction of molecules, and introduce some advanced analysis tools such as different filters,^(^^94^ ^)^ freehand cluster drawing, cluster analysis with Machine Learning, FRET trajectory estimation, and calculation of absolute concentration of NADH.

## Supporting information

Gottlieb et al. supplementary material Gottlieb et al. supplementary material

## Data Availability

FLUTE is Copyright (C) 2022 FLUTE. FLUTE source code is Free and Open-Source Software released under the terms of the 3-Clause BSD License. The prebuilt FLUTE executable is a combined work that contains both FLUTE and QT library bindings. It is released under the terms of both the 3-Clause BSD License (for the FLUTE part) and the GNU Lesser General Public License (for the QT part). Source code and executable are available on the GitHub repository at https://github.com/LaboratoryOpticsBiosciences/FLUTE. The FLIM data relative to the publications are in the Zenodo repository at https://zenodo.org/record/8324901.

## Supplementary material

The supplementary material for this article can be found at http://doi.org/10.1017/S2633903X23000211.
