# Supplementary material for: FLUTE: A Python GUI for interactive phasor analysis of FLIM data
Source: Biol Imaging. 2023 Nov 6;3:e21. doi: 10.1017/S2633903X23000211 (PMC10936343; doi:10.1017/S2633903X23000211)
Supplement: Gottlieb et al. supplementary material [file S2633903X23000211sup001.pdf]

Supplementary Information and User Manual

FLUTE

Fluorescence Lifetime UlTimate Explorer

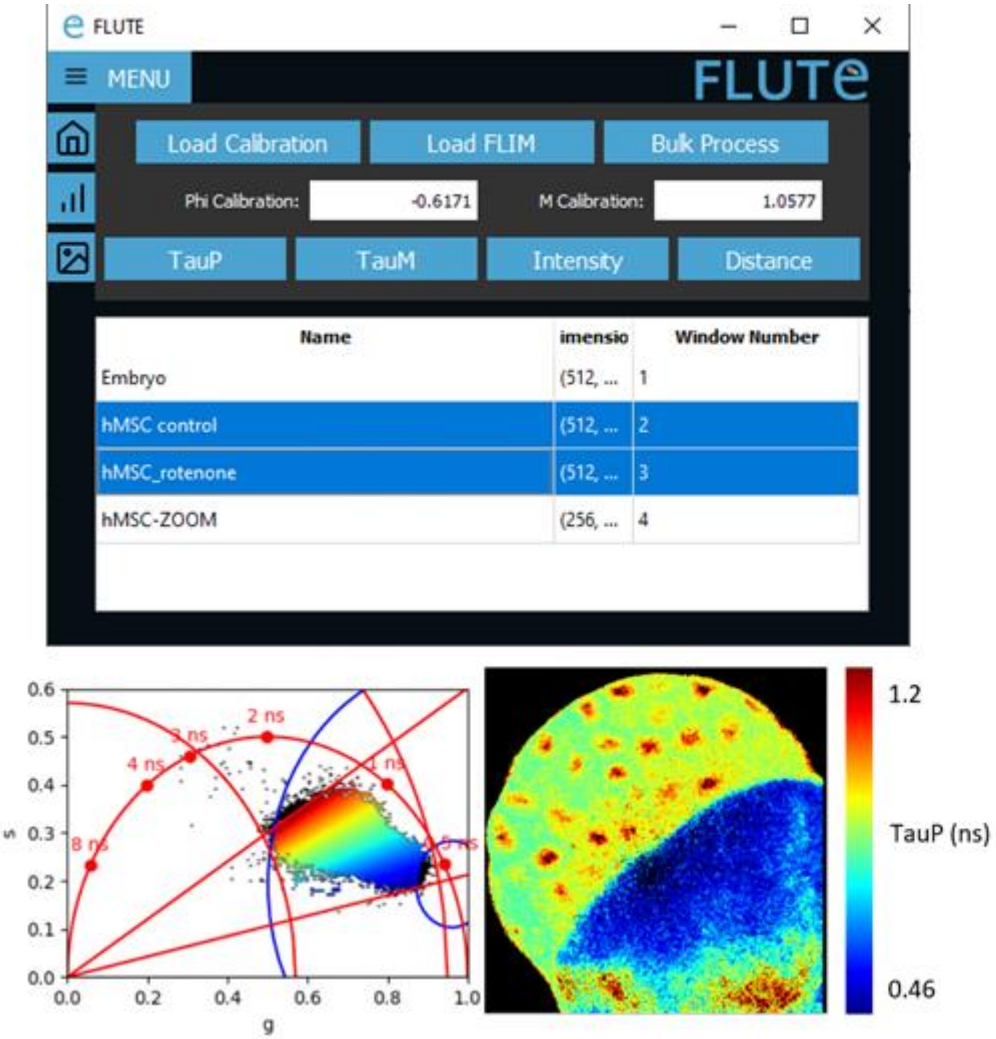

Table of contents

1. Running FLUTE 3
   1.1 Run exe file 3
   1.2. Run main.py 3
2. Main window navigation 4
3. Calibration with a lifetime reference 5
4. Loading FLIM data 6
5. Selecting datasets using the table 7
6. Median filter 7
7. Intensity filter 8
8. Changing color maps 8
   8.1 TauP color map 9
   8.2 TauM color map 10
   8.3 Distance color map 10
   8.4 Circle selection 11
9. Interactivity window 11
   9.1 Range adjustment 12
   9.2 Closing datasets 12
   9.3 Saving data 13
10. Batch processing 14
11. Converting FLIM data in different formats 14
   11.1. Conversion of Becker & Hickl (.sdt) files to .tiff files 15
   11.2. Conversion of ISS (.fbd) files to .tiff files 16
   11.3. Conversion of Picoquant (.ptu) files to .tiff files 17
12. Comparison between FLUTE and other free and open-source software tools for Phasor analysis 20
13. Processing time measurement 21
14. References 21

1. Running FLUTE

FLUTE can be run in two different ways.

1.1 Run exe file in Windows operating system

Download FLUTE.exe from GitHub (https://github.com/LaboratoryOpticsBiosciences/FLUTE) and double-click FLUTE.exe.

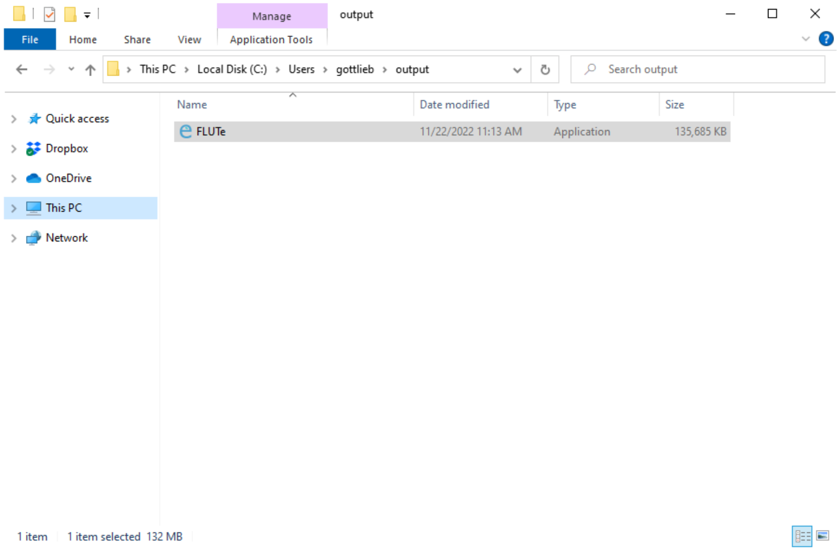

Figure S1: *Running FLUTE.exe*

1.2. Run main.py in different operating systems

If Python is installed on your system, open the terminal; install all the necessary packages: *"pip install PyQt5, numpy, opencv-python, matplotlib, scikit-image"*; navigate to the folder containing main.py and type: python main.py.

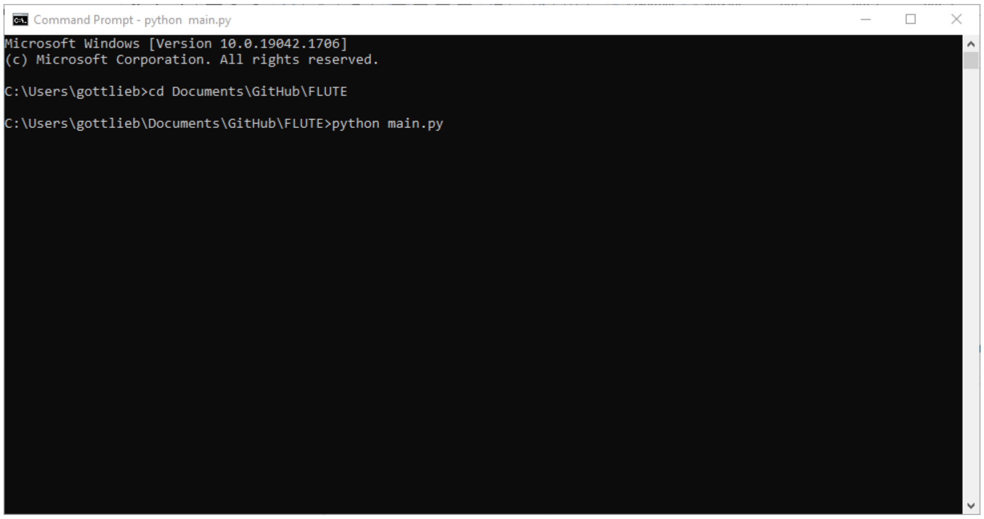

Figure S2: *Running main.py using a computer with Python installed*

Running 'main.py' has been tested to work on different Operating systems: Windows, Linux (with Ubuntu 20 and 22.04) and MacOS including M1 and M2 chips.

Here we describe an easy procedure to run main.py with a Mac and/or Windows by automatically downloading all the necessary files from FLUTE's GitHub page:

a) Install Anaconda Navigator, which is a GUI to manage Python environments and libraries. The documentation is available here: https://docs.anaconda.com/free/navigator/index.html
b) Go to the Environments tab and create a new environment for running FLUTE. It is not mandatory, but much recommended, because it will help you to avoid library dependencies in case if you use Python to run other programs.
c) In the same Environments tab with the FLUTE environment activated, on the top right corner you can search and install specific versions of libraries. Install necessary packages into new environment with the following command*: "pip install PyQt5, numpy, opencv-python, matplotlib, scikit-image"*
d) Install PyCharm Community edition (either from Anaconda homepage or online), then upon startup you can just insert a link to the FLUTE's GitHub page https://github.com/LaboratoryOpticsBiosciences/FLUTE and all necessary files will download automatically. Then go to Preferences -> Project: main.py -> Python Interpreter, and select Anaconda environment you've created.
e) Run main.py

Here we describe a troubleshooting procedure to run 'main.py' on Linux Operating systems**.**
After installing all dependencies (PyQt5, matplotlib, numpy,opencv-python and scikit-image) with the "pip install" command, the program "python main.py" may fail with the message :
qt.qpa.plugin: Could not load the Qt platform plugin "xcb" in "/home/lambry/.local/lib/python3.10/site-packages/cv2/qt/plugins"
This application fails to start because no Qt platform plugin could be initialized. Reinstalling the application fixes this problem. This problem is solved with the commands: *pip uninstall opencv-python pip install opencv-python-headless.*

2. Main window navigation

Figure S3 shows how the main window is divided into several sections, referred to as **Home**, **Interactivity**, **Thresholding**, and **Table** for the remainder of this document. The names of the windows are accessed by pressing the **Menu** button.

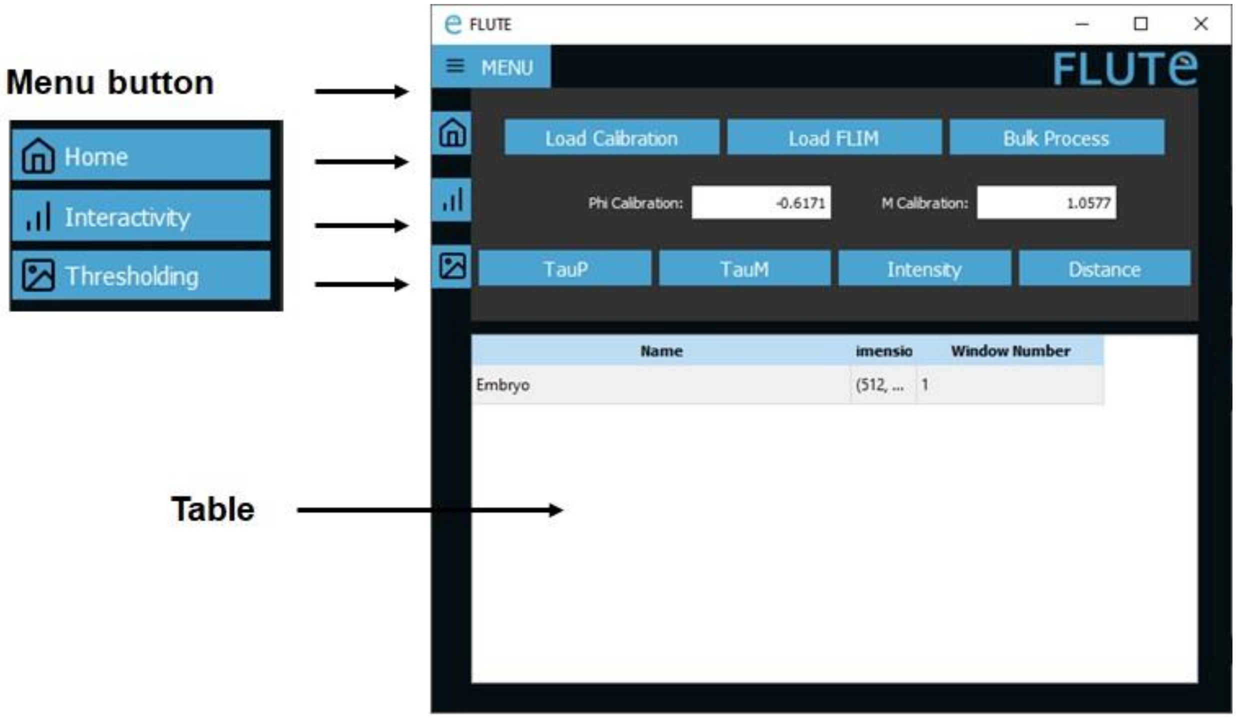

Figure S3: *labeled windows for FLUTE main user interface. On the left, there are the Home window, Interactivity window, and Thresholding window buttons. Table displays the list of loaded data.*

3. Calibration with Lifetime reference

To calibrate FLIM data with FLUTE, click **Load Calibration** on the **Home** window and enter the setup parameters in the popup window:

- Bin Width (ns): Duration of a single temporal bin of the time-domain FLIM acquisition
- Laser Freq. (MHz): Laser repetition rate
- Tau Ref. (ns): Known lifetime of the single-exponential reference sample (e. g. 4 ns for fluorescein)
- Harmonic: Integer multiple applied to laser frequency that will be used to calculate the Fourier Transform

FLUTE calculates the **Phi Calibration (**$\Delta\varphi$**)** and **M Calibration (**Δm **)** values (equations 9 and 10 of main text) to be applied for calibration and displays them in the home menu as shown in Fig. 8. An example of fluorescein decay is uploaded on our GitHub repository [1] as *Fluorescein.tiff* stack. After calibration if you load the same calibration file (*Fluorescein.tiff*) it is now located at the correct location of 4 ns in the phasor plot (Fig. S4). The same **Phi Calibration (**$\Delta\varphi$**)** and **M Calibration (**Δm **)** values will be applied to all the FLIM data opened with the **Load FLIM** function.

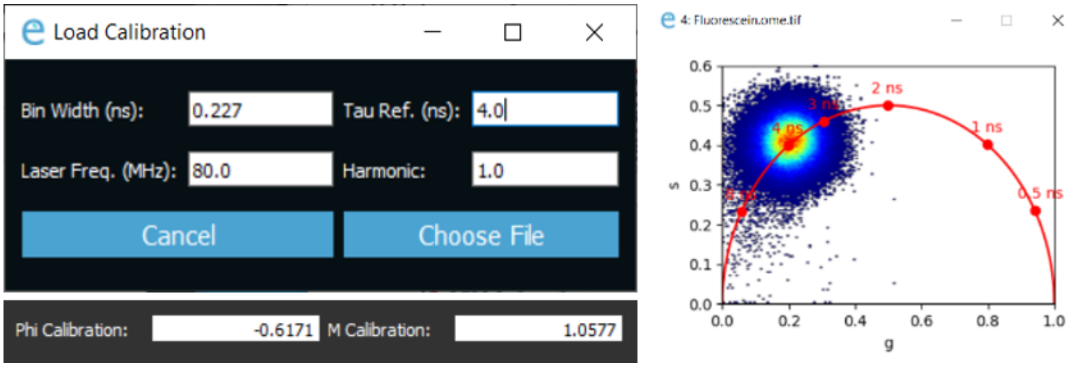

Figure S4: *Calibration windows of FLUTE and example of calibrated experimental fluorescein sample with 4 ns lifetime.*

With FLUTE it is possible to perform Phasor analysis at higher frequency of the laser repetition rate, by changing the “Harmonic” parameter in the “Load Calibration” window. An example of calibration with a 0 ns lifetime SHG starch sample (*starch SHG-IRF.tiff in* our GitHub repository [1]) is shown in Figure S5: the FLIM data set *ZF-1100_noEF.tiff* is phasor transformed with the first harmonic of the laser repetition rate, i.e. 80MHz (Fig. S5a) and with the second harmonic of the laser repetition rate, i.e. 160MHz (Fig. S5b).

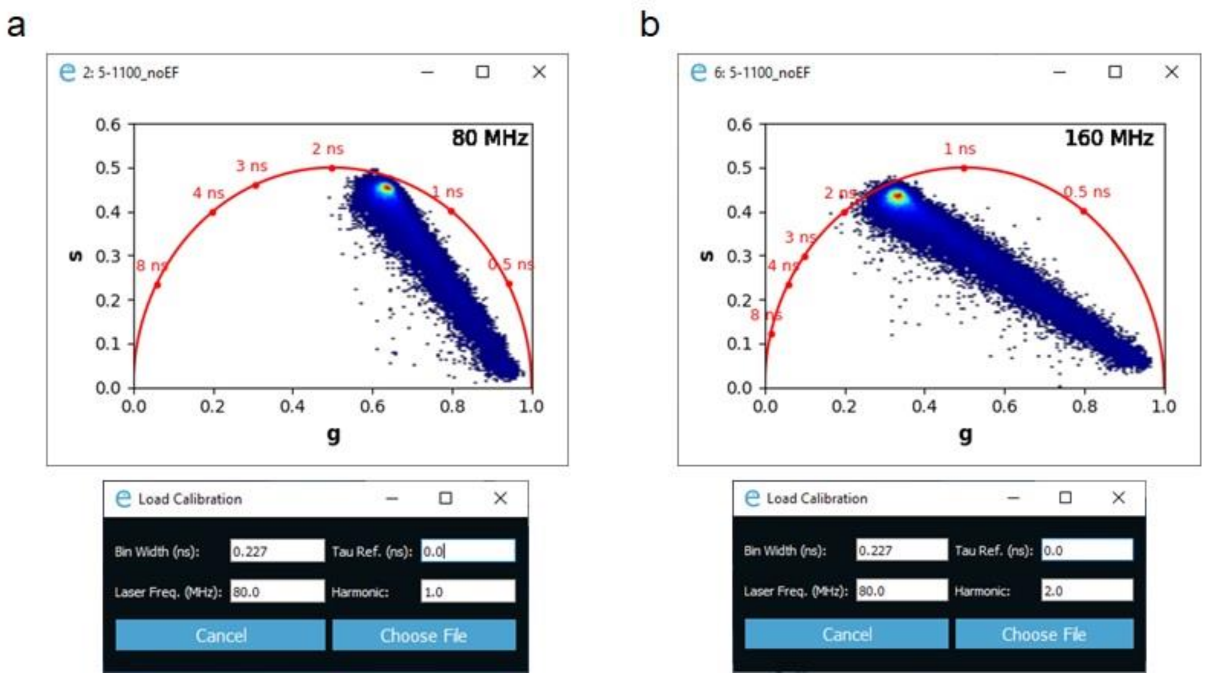

Figure S5: *Calibration of FLIM image (ZF-1100_noEF.tiff) using the SHG at 0 ns lifetime (starch SHG-IRF.tiff) as a calibration. Phasor transformation and plot are calculated at the first (A) and second (B) harmonic of the laser repetition rate.*

4. Loading FLIM data

In the **Home** window, press **Load FLIM**, and select the data to open. FLIM data must be uploaded as a .tiff stack. You can use the shortcut ctrl+click or shift+click to select multiple datasets at once. The phasor plot with a histogram density color map and intensity image will open for each dataset, and the table will be

populated. Figure S6 shows the example of *Embryo.tif* FLIM file that can be found in on our GitHub repository.

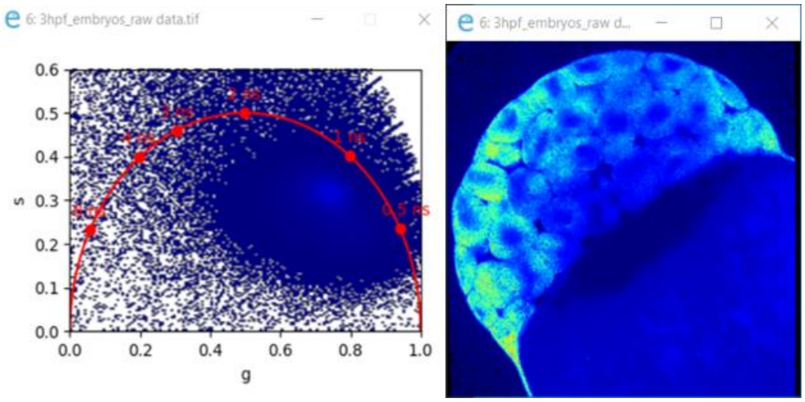

Figure S6: *Loaded Embryo.tif data before any changes are applied.*

5. Selecting active datasets using the table

In all following sections, changes to datasets are only applied when the data is active and selected in the table. To select data, either click whichever is desired, or ctrl+click or shift+click to select multiple sets of data. In the example provided in figure S7, changing parameters such as color maps or intensity threshold will only apply to windows 1, 3, and 4, but will not be applied to windows 2 and 5.

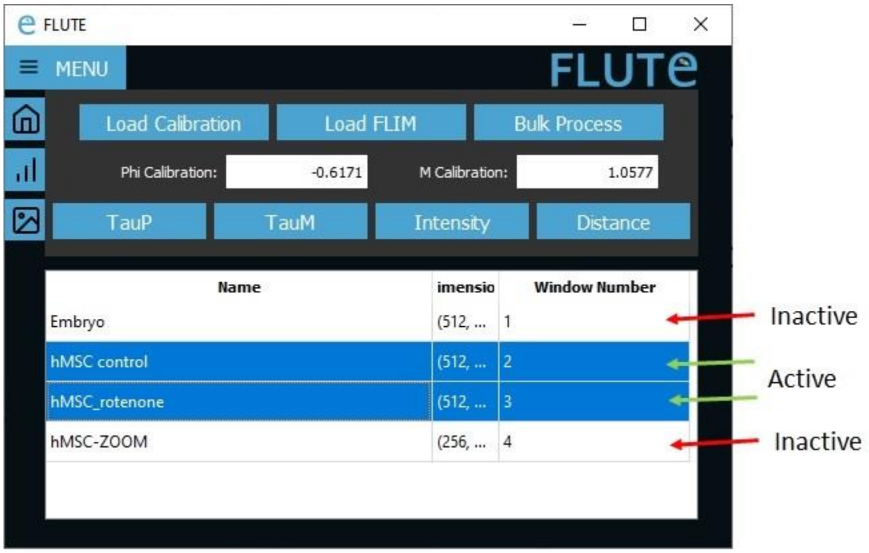

Figure S7*: Selecting active and inactive data in the Table on the Main Window.*

6. Median filter

Successive median filters can be applied to the g and s matrices using the **Median Filt** entry box within the **Thresholding** window. A 3x3 convolutional median filter is applied n times to the s and s coordinates of the phasor plot using scipy.signal.medfilt. Figure S8 displays the use of 3 median filters applied to the *Embryo.tif* FLIM file of a Zebrafish embryo at three hours post fertilization (as for now referred to as 3hpf):

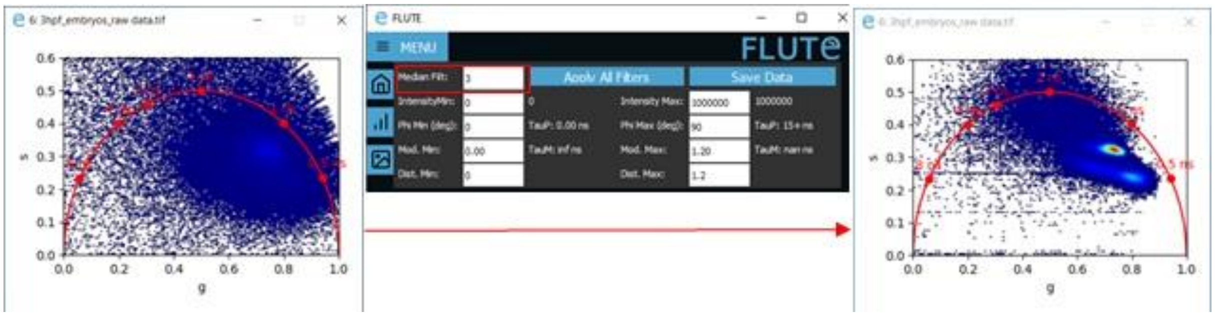

Figure S8: *Applying 3x3 convolutional median filter to data.*

7. Intensity filter

Minimum and maximum intensity thresholds can be applied using the **IntensityMin** and **IntensityMax** entry boxes inside the **Thresholding** window. The intensity is calculated as the sum of all images in the .tiff stack. As shown in figure S9, applying a min intensity threshold of 25 counts to the 3hpf dataset removes the background:

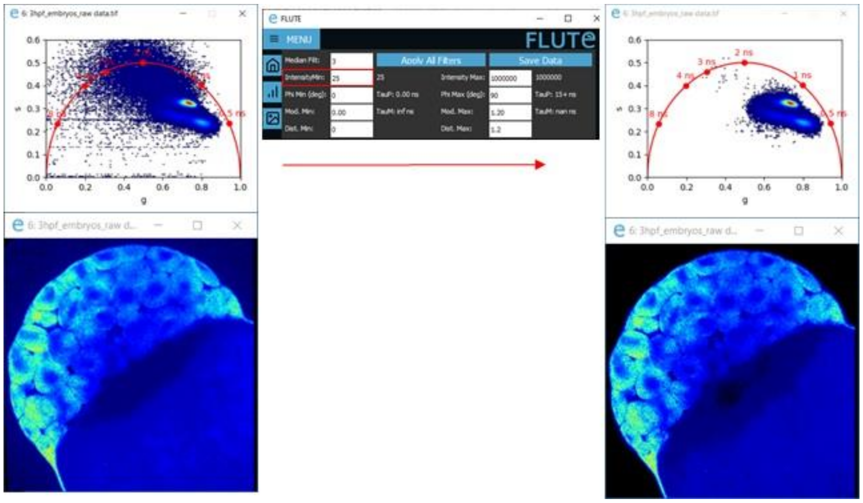

Figure S9: *Applying Intensity threshold to FLIM data.*

8. Changing color maps

FLUTE gives to the user the flexibility to choose the appropriate color mapping to be applied to the FLIM image and the phasor plot simultaneously. For FLIM data exploration and visualization the image window is switchable between the options presented in figure S10.

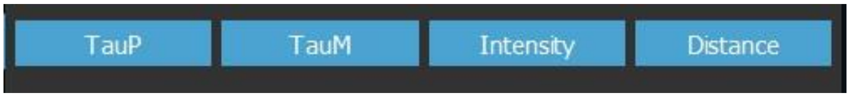

Figure S10*: Image visualization options: intensity, lifetime contrasts and distance*

As seen in figure S10, available color maps are:

- <u>TauP</u>: Phase lifetime calculated with equation 7
- <u>TauM</u>: Modulation lifetime calculated with equation 8

- Intensity: Based on the intensity of the pixel across the entire tiff stack. Both a jet and a greyscale color maps are available for intensity. Clicking the **Intensity** button will switch between the two.
- Distance from a molecular species.

The threshold of the relative color maps can be adjusted either via the **Interactivity window**, according to figure S11:

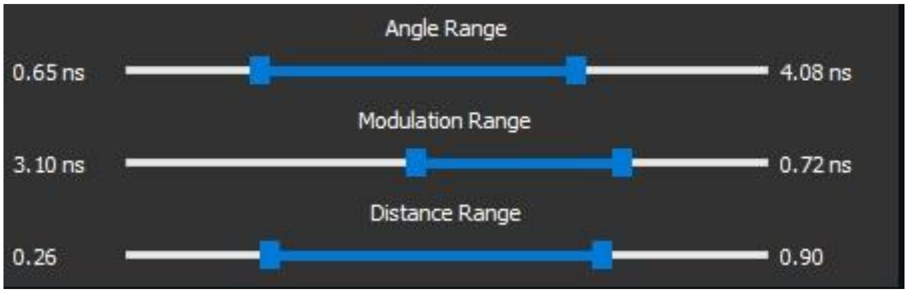

Figure S11: *Thresholding range sliders available in the Interactivity window.*

Or, via the **thresholding window**, shown in figure S12:

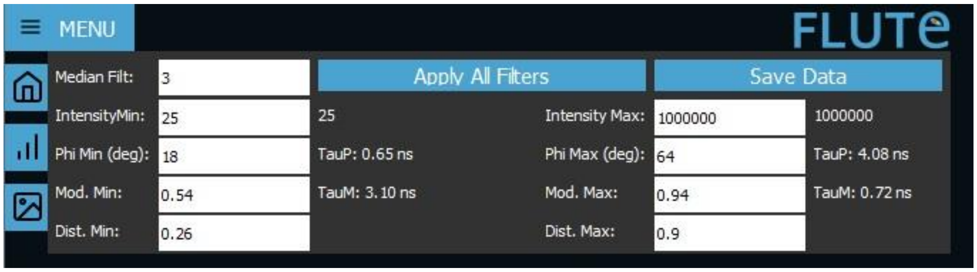

Figure S12: *Thresholding entry boxes available in the Thresholding window.*

8.1 TauP color map

TauPhase (TauP) is calculated with equation 8. Applying the TauP color map to the data and setting the **Phi Min (deg)** and **Phi Max (deg)** values to 12 and 35 degrees in the **Thresholding** window respectively will result in the image presented in figure S13.

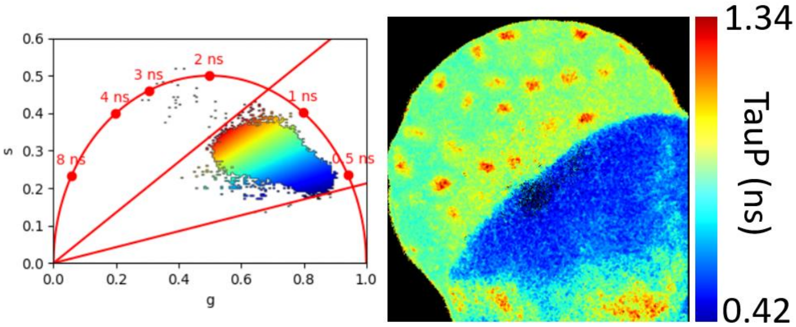

Figure S13: *FLIM data mapped with TauP contrast.*

8.2 TauM color map

TauModulation (TauM) is calculated with equation 9. Applying the TauM color map to the data and setting the **Modulation Min** and **Modulation Max** values to 0.59 and 0.92 in the **Thresholding** window respectively will produce the image presented in figure S14.

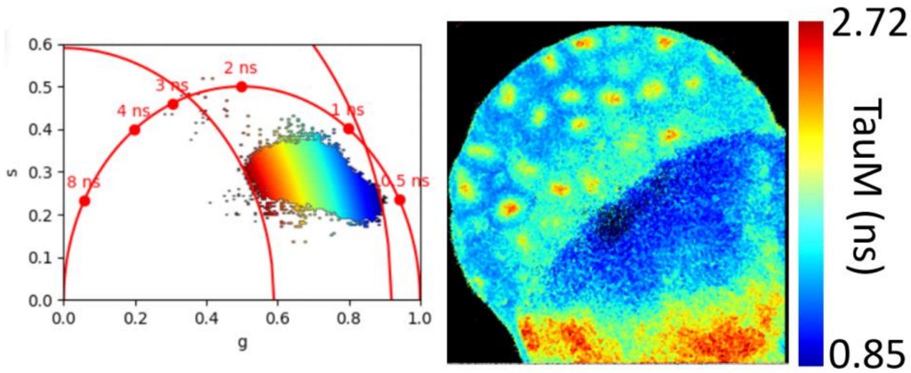

Figure S14: *FLIM data mapped with TauM contrast.*

8.3 Distance color map

FLUTE can be used to calculate and map the distance from the molecular species B by inserting the known phasor coordinates of species B. When the Distance color map is selected, a popup window (figure S15) appears with two options: insert the single lifetime of the fluorophore or the g and s phasor location of the molecule in the phasor plot.

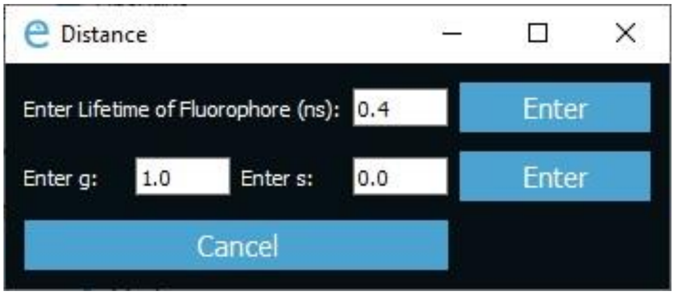

Figure S15: *Entry box to apply options for distance color mapping*

Enter Lifetime of Fluorophore (ns) takes in account the phasor coordinates of the molecular species B either inserting the single exponential lifetime or the phasor coordinates g and s in the case of a multi-exponential decay. FLUTE then calculates the distance from molecular species B by using equation 15. For example, the distance from free NADH can be calculated graphically and mapped in every pixel from the location of the free NADH that has a known single lifetime of 0.4 ns. Figure S16 displays the application of the distance from free NADH color map in the *Embryo.tif FLIM* file.

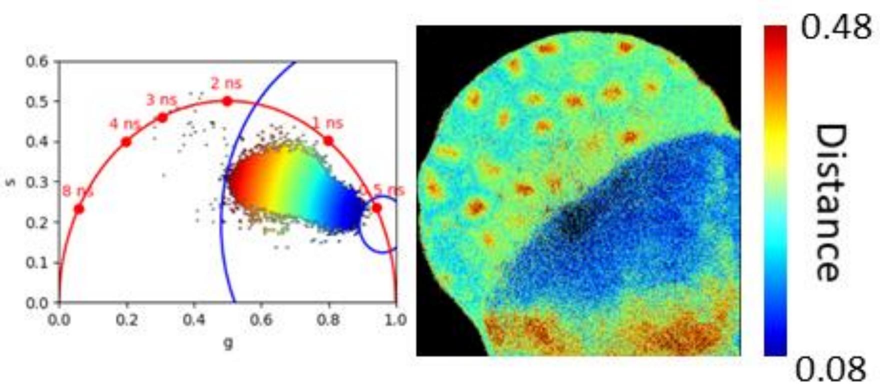

Figure S16: *FLIM Data mapped with the distance from free NADH contrast.*

8.4 Circle selection

Interactive exploration of the FLIM data can be performed by using multiple colored cursors of variable sizes to select pixels with similar decays and to highlight simultaneously the corresponding pixels in the image. Areas on the phasor plot can be selected by clicking on an active plot. The modification of color and size is done through the **Interactivity** window using the **Color** and **Radius** options. In figure S17, the embryo image is highlighted with a red circle of radius 0.05, and a green circle of radius 0.1, with a greyscale color map.

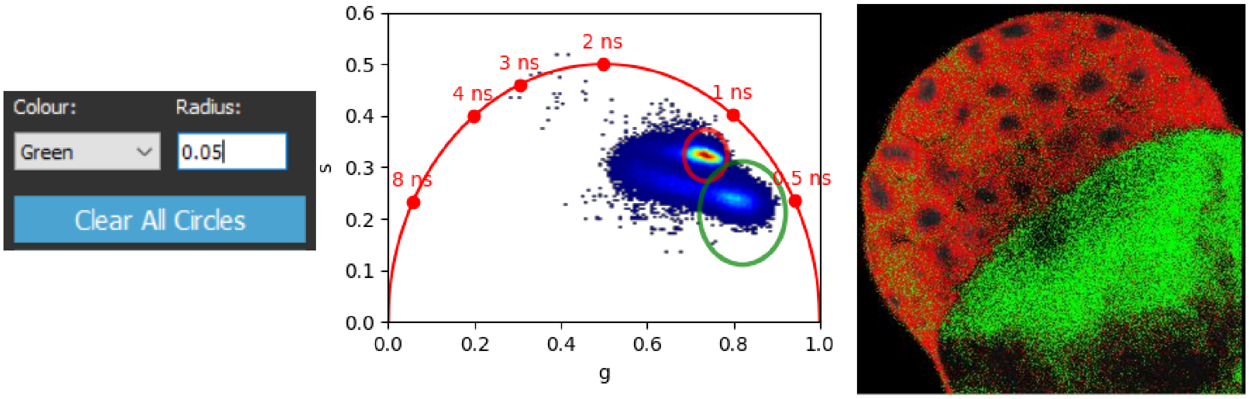

Figure S17: *Data with intensity color mapping and regions selected with a red circle of radius 0.05 and a green circle of radius 0.1.*

Clicking **Clear All Circles** removes all circles from the plot.

If there are multiple active datasets, clicking on one active phasor plot will draw the same circle on all other active phasor plots.

9. Interactivity window

The **Interactivity** window is used for quick and coarse thresholding of the FLIM data and interactive data exploration.

9.1 Range adjustment

Through the interactivity window we can apply a threshold to the phase (φ), modulation (m), and distance range simultaneously using the sliders as displayed in figure S18. For example, the phasor of the *Embryo.tif* FLIM image in (figure S18b) has been applied different parameter thresholds that are represented by the colored lines (red for phase and modulation and blue for distance). The thresholded pixels are represented in black in the phasor plot.

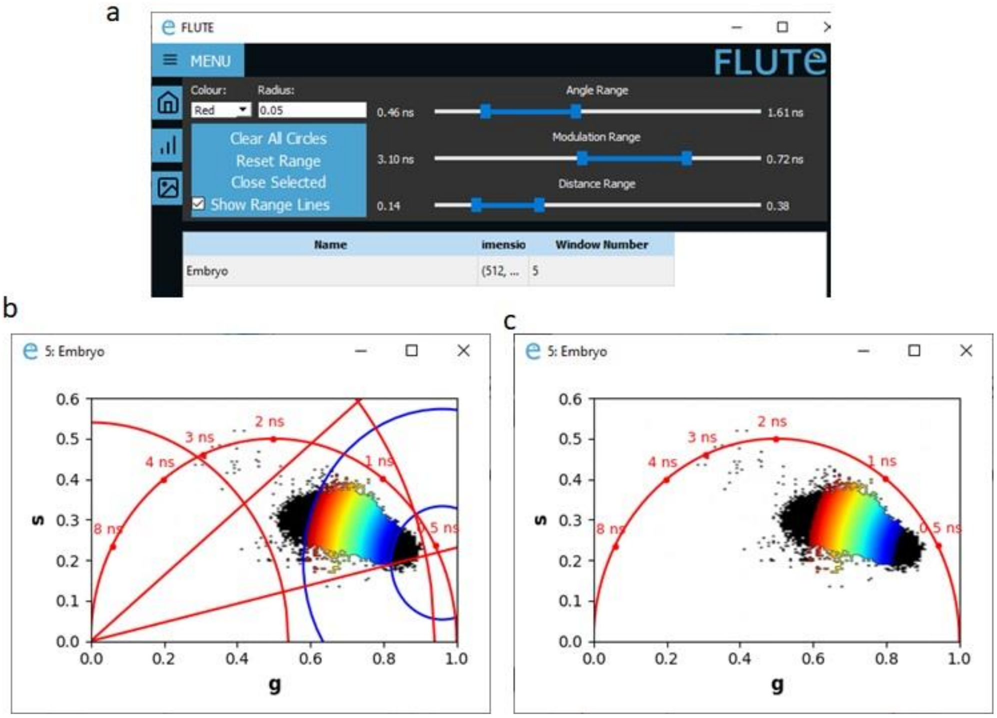

__Figure S18:__ Range adjustment thought the interactivity window (a) Overview of all available thresholds applied to the data. (b-c) Phasor plots with applied thresholds on the different parameters (phase and modulation and distance with (b) and without (c) colored lines that represent the thresholds.

Clicking **Reset Range** function (Fig.18a) will remove all the applied threshold modifications and will reset the range of the parameters to the default values.

Unclicking **Show Range Lines** function (Fig.18a) will remove all the threshold lines from the phasor plot, while keeping the applied threshold to the FLIM data and phasor cloud (Fig.18c).

9.2 Closing datasets

Closing multiple active datasets is possible by choosing all in the table and clicking **Close Selected**, as shown in figure S19.

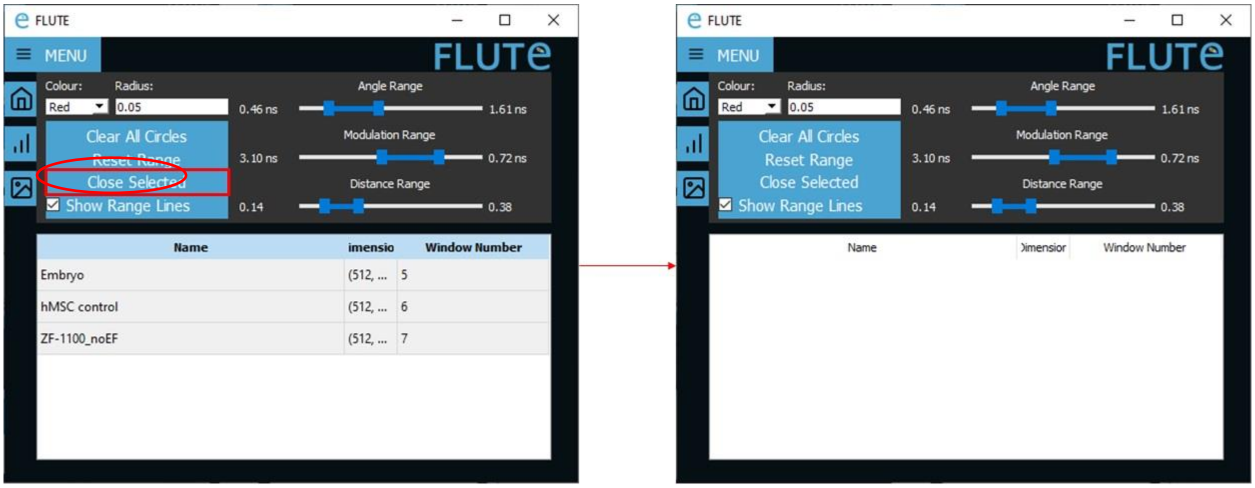

Figure S19: *Closing multiple sets of data at once.*

9.3 Saving data

In addition to fast and interactive FLIM data visualization, analyzed data with applied parameter thresholds can be saved in FLUTE using the Save Data button. Clicking Save Data is followed by a popup window that can save either all the color maps (All Data) or the color map that is on display (Currently Displayed). Saved data is listed below; also, an example is given in figure S20.

- …_g.tiff, …_s.tiff: the g and s coordinates with applied parameter thresholds. Thresholded pixels are saved as ‘nan’. Data are saved as a 32bit floating point tiff files.
- …_TauP.tiff, …_TauM.tiff, …_Dist.tiff: The TauP lifetime, TauM lifetime, and Distance values of the dataset with the applied parameter thresholds. Data is saved as a 32bit floating point tiff file with thresholded values saved as ‘nan’.
- …_graph_density.png, …_graph _Distance.png, …_graph _TauM.png, …_graph _TauP.png: Phasor plot screenshots for the various color maps.
- …_imageDistance.tif, …_imageIntensity.tif, …_imageJet.tif, …_imageTauM.tif, …_imageTauP.tif: screenshots of the FLIM imageswith the respective color maps with parameter thresholds applied . Data are saved as RGB.
- …_Parameters.txt: List of parameters thresholds applied to the dataset and average values of the phasor coordinates g and s, tauP, tauM and distance measured in the mask.

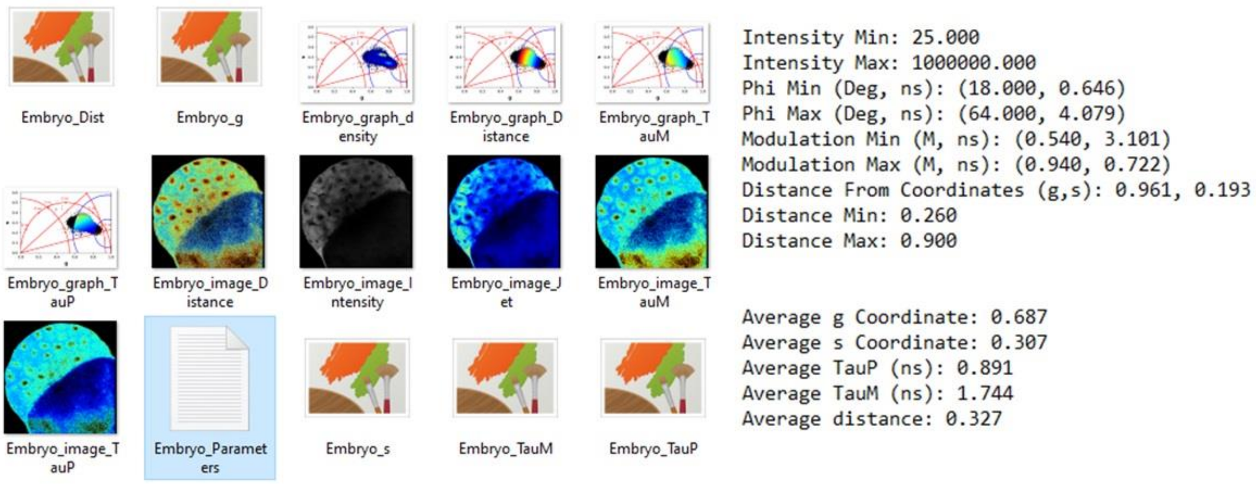

Figure S20: *Example of saved files (left) and parameter file (right)*

10. Batch processing

FLUTE has been designed for bulk processing, which is useful for the fast analysis of multiple FLIM data from a single experimental session.

To perform bulk processing, first apply the desired thresholding values in the **Thresholding** window. On the **Home** window, click **Bulk Process**. In the first popup window (figure S21), select the desired data to be processed using ctrl+click or shift+click, and click Open. In the next window, select a folder to save the data. FLUTE will open all datasets; perform the phasor transformation, apply the indicated filters and thresholds, and save all the output data.

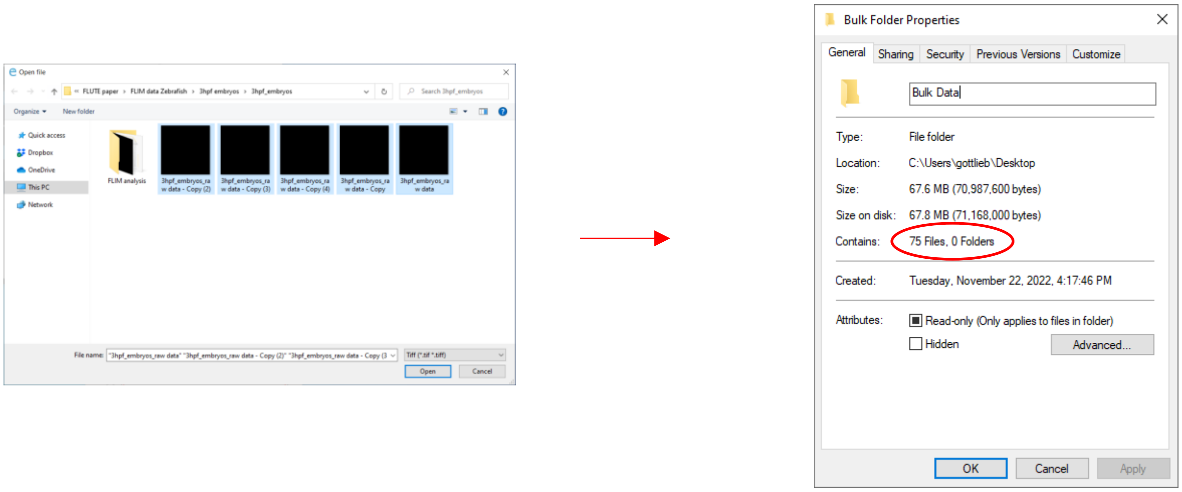

Figure S21: *Batch processing five images leads to 75 saved data files inside the selected folder*

11. Converting FLIM data in different formats

With the current version of FLUTE, FLIM data acquired with commercial cards have to be first converted into a .tif stack using either the software of the company or available open sources plugins. Briefly: .std

FLIM formats from Becker & Hickl can be easily transformed in .tif stacks by using the open-source Bio-Formats [2] plugin for ImageJ; .fbd files from ISS can be exported into .tif files from The VistaVision software (See Supplementary Information 11.2); .ptu files from Picoquant can be exported into .tif files using first SymPhoTime64tware software to extract the binary .bin file and then files using the open-source plugin Bio-Formats toolbox to convert the .bin file in .tif or directly exporting .ptu files in to .tif files using the open-source plugins PTU_Reader for ImageJ [3] (See Supplementary Information 11.3).

11.1. Conversion of Becker & Hickl (.sdt) files to .tiff files.

.std files from Becker & Hickl can be exported into .tiff files using the opensource plugin Bio-Formats toolbox [2]:

a) Make sure that you have the Bio-Formats plugin installed in ImageJ.
b) Open the desired .sdt file with ImageJ panel, a Bio-Formats window will appear automatically (Fig S22).
c) Check the case "Display metadata".
d) The stack will open in ImageJ together with the related metadata file
e) Save the FLIM stack in .tif format.
f) From the metadata file extract the acquisition parameters (Fig. S23): *"Size T"* (line 10) indicates the number of temporal bin. while if *"MeasStopInfo.maxSyncRate"* (line 146) is equal to *"MeasStopInfo.minSyncRate"* (line 150) these values indicate the laser repetition rate.
g) Calculate the value of the bin width: *bin width =1/ MeasStopInfo.maxSyncRate/Size T*

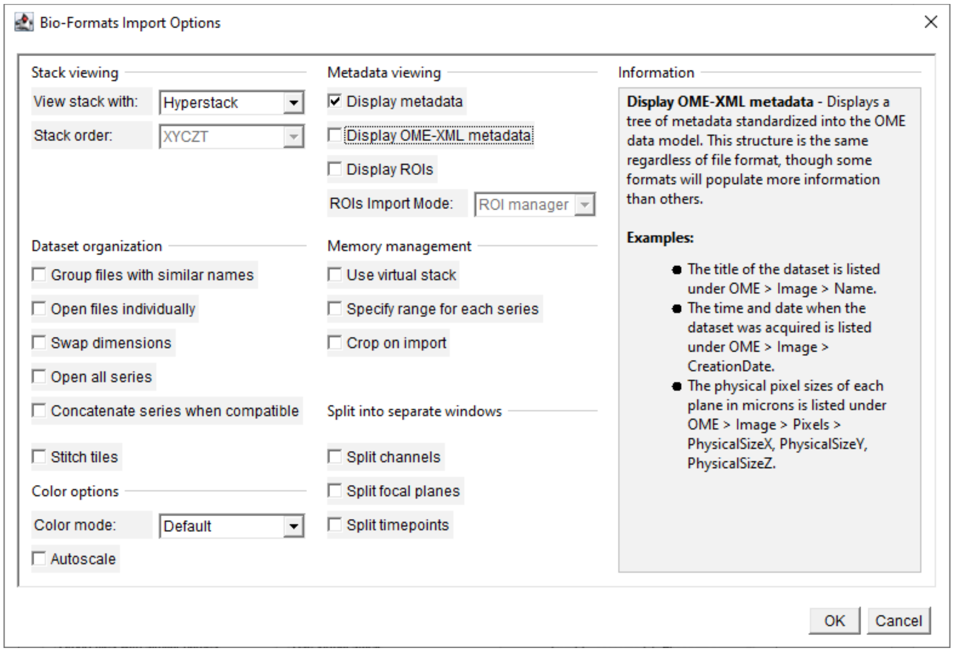

Figure S22: *Bio-formats Import Options Panel*

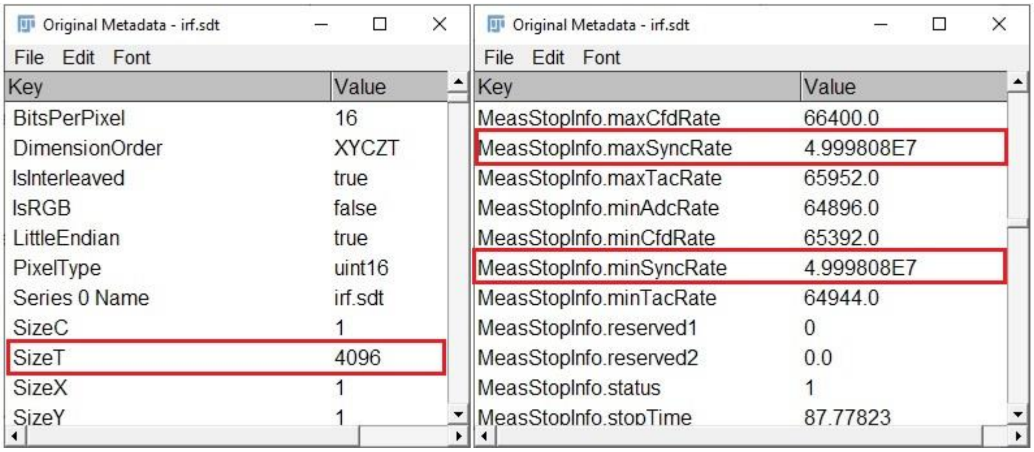

Figure S23: Extracting acquisition parameters number of temporal bin number and laser repetition rate from the metadata of the .std file

In the future version of the FLUTE code we aim to integrate the file reading of the .std file inside the Python code to provide a more compact version of the analysis that does not require additional steps with other external software. With this goal we aim to use the Python library "sdtfile" developed by Christoph Gohlke to read Becker & Hickl .std files [4, 5].

11.2. Conversion of ISS (.fbd) files to .tiff files

.fbd files from ISS can be exported into .tiff files from The VistaVision software (See also VistaVision manual pages 28-29)

a) Click the icon under the decay plot to open che *"Export Data"* dropdown list, from which the user can export the raw decay data (Fig. S24).
b) Click the "*Export Frame Time Decay Raw Data"* tab to export the photon counts per time bin, as a single TIFF image file containing a series of images.
c) The number of images contained in the signgle TIFF image file is the same as the number of the time bins specified by the TD resolution. The i-th image in the sequence represents the corresponding time bin along the time decay
d) When you open the .fbd file, make sure the supporting files (ifil, xlm) with the same file name is in the same folder as the .fbd. These supporting files are usually automatically generated when the images are saved.
e) From the metadata .xlm file (Fig. S25) extract the acquisition parameters: "*Phase*" that indicates the number of temporal bins and "*Excitation Frequency*" that indicated the laser repetition rate
f) Calculate the value of the bin width: *bin width =1/ Excitation Frequency /Phase*

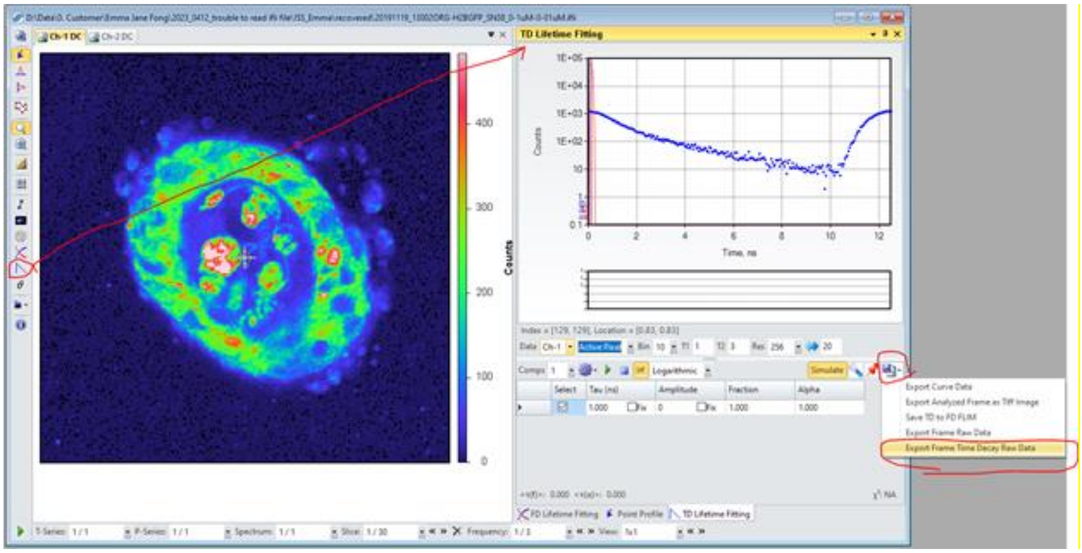

Figure S24: *Screenshot of the VistaVision software to Export data*

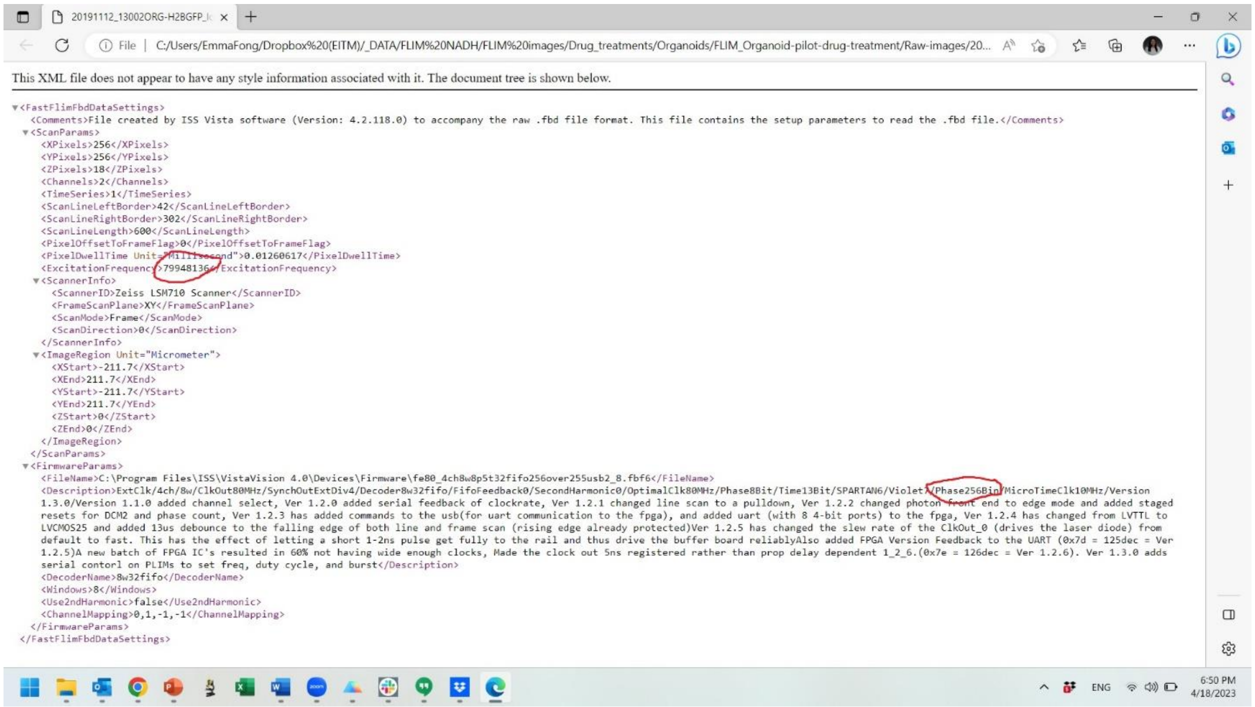

Figure S25: Screenshot of the .xlm file. Extracting acquisition parameters number of temporal bin number and laser repetition rate from the metadata of the .fbd file

11.3. Conversion of Picoquant (.ptu) files to .tiff files.

.ptu files from Picoquant can be exported into .tif files using first SymPhoTime64tware software to extract the binary .bin file and then files using the opensource plugin Bio-Formats toolbox to convert the .bin file in .tif [2]:

a) In SymPhoTime64tware software click the *"Export Binary"* button to generate a specific binary file .bin from the .ptu file (Structure of the binary file is shown in Fig S.26)

b) Make sure that you have the Bio-Formats plugin installed in ImageJ.
c) Open the binary .bin file with ImageJ and a Bio-Formats window will appear automatically (Fig S21).
d) Check the case *"Display metadata"*.
e) The stack will open in ImageJ together with the related metadata file.
f) Save the FLIM stack in .tiff format.
g) The acquisition parameters *"Size T"* indicates the number of temporal bin. while if *"TTResult_syncRate"* (line 49) indicates the laser repetition rate and can be extracted from the metadata file or the header of the ptu file (Fig S27).
h) Calculate the value of the bin width: *bin width =1/ MeasStopInfo.maxSyncRate/Size T*

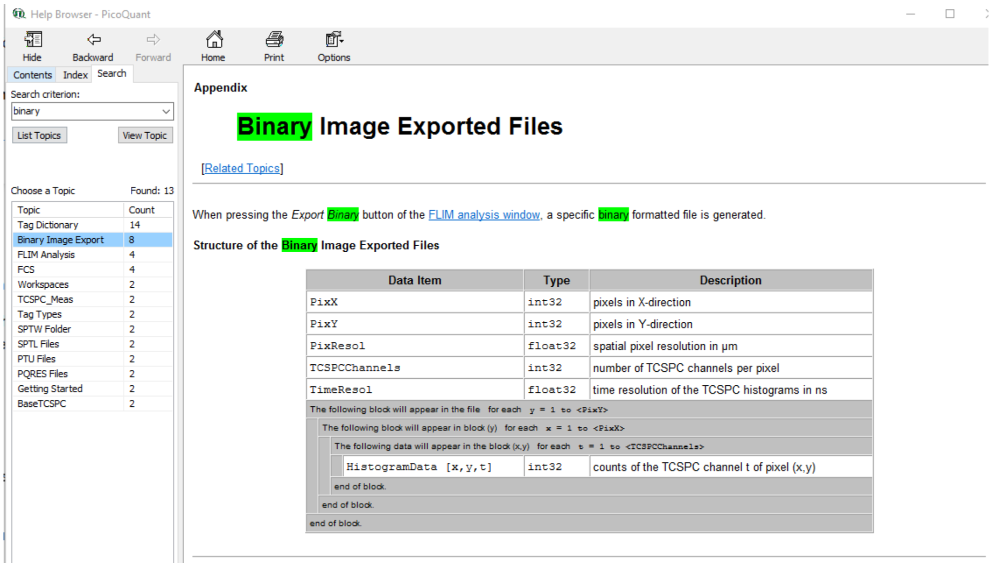

Figure S26: Screenshot of the structure of the binary file .bin is displayed. It is possible to find the same information in the following webpage: https://www.tcspc.com/doku.php/glossary:pre-histogrammed_image

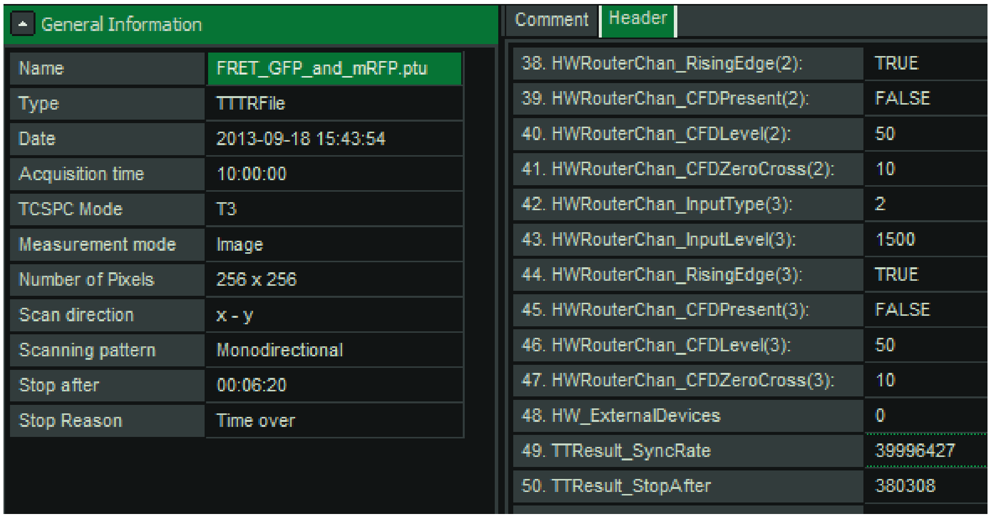

Figure S27: Screenshot of the .ptu file header containing the metadata with the acquisition parameters *"Size T"* and *"TTResult_syncRate"*.

Alternatively .ptu files from Picoquant can be exported directly into .tiff files using the open source plugins PTU_Reader for Image J.[3]:

1. Go to Plugins > PTU reader, navigate to the file.
2. The data load parameters window will appear:

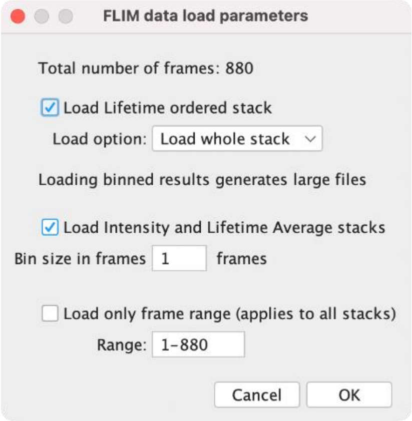

Figure S28: Screenshot of the PTU_Reader FLIM load parameters

3. Also the file metadata will appear:

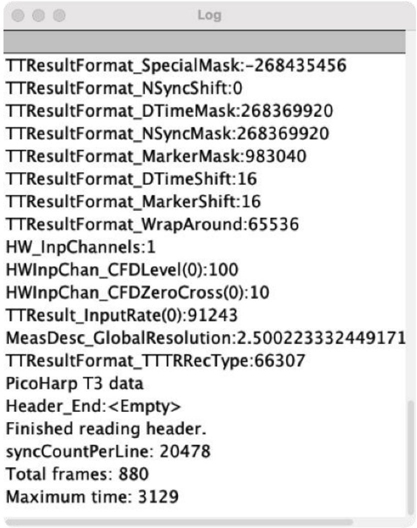

Figure S29: Screenshot of the file metadata opened with PTU_Reader

In the future version of the FLUTE code we aim to integrate the file reading of the .ptu file inside the Python code to provide a more compact version of the analysis that does not require additional steps with other external software. With this goal we aim to use the Python code *"Read_PTU.py*" developed by Picoquant to read .ptu files [6] or *"readPTU_FLIM"* developed by the Brinks Lab [7].

12. Comparison between FLUTE and other free and open-source software tools for Phasor analysis.

Software	SimFCS	PAM	FLIMJ	FLUTE
Relative publication	Ranjit et al. 2018	Schrimpf et al. 2018	Gao et al. 2020	
Open source code	No	Yes	Yes	**Yes**
Free	Yes	No	Yes	**Yes**
Programming language	C++	MATLAB	Java	**Python (facilitates broad use and extensibility)**
OS availability (Windows, Linux, MacOS including M1 and M2 chips)	Yes	Yes	Yes	Yes
Preprocessing features	Median filter (in preprocessing stage), intensity thresholding	Median filter (in preprocessing stage), intensity thresholding	Yes (filters available on ImageJ	Median filter (reversible application), intensity thresholding
FRET trajectory	Yes	Yes	No	No
**Interactive visualization**				
Manual cluster analysis	Yes	Yes	No	Yes
tauP and tauM display	No	Yes (not interactive)	Yes (not interactive)	**Yes (interactive)**
Mapping distance/fraction of molecular species	Yes (manual drawing)	Yes (manual drawing)	Yes	**Yes (by defining species phasor location-facilitates reproducibility)**
Interactive changing colormap of phasor and image	No	No	No	**Yes (with thresholding entry boxes or range sliders)**
Calculating the absolute NADH concentration	Yes	No	Scriptable endpoint through ImageJ-Ops	No
Measuring mean in ROI	Yes	Yes	Yes	Yes
Batch processing	Yes	Yes	Scriptable and scalable through ImageJ-Ops	Yes

Table 1. Comparison between FLUTE and other free and open-source software tools for Phasor analysis.

13. Processing time measurements

Here we estimate the average processing time for a phasor transformation and the analysis time per image in the bulk process that includes also the applications of filters, measurements and saving the images and the results. The measurements were performed on images of 256 x 256, 512 x 512 and 1024 x 1024 pixels with a MacBook Air (M1, 2020) with a Chip Apple M1 and 8 GB Memory.

Summary

**Image dimension** (pixel x pixel)	**Phasor transformation time** (s)	**Analysis time in blulk process** (s per image)
256 x 256	~0.7	~1.8
512 x 512	~1.1	~2.5
1024 x 1024	~3.0	~5.4

Table2. Estimation of processing times for individual phasor transformation and FLIM image analysis in the bulk process

14. References

1. *https://github.com/LaboratoryOpticsBiosciences/FLUTE.*
2. *https://www.openmicroscopy.org/bio-formats/.*
3. *https://github.com/UU-cellbiology/PTU_Reader.*
4. *https://github.com/cgohlke/sdtfile.*
5. *https://pypi.org/project/sdtfile/.*
6. *https://github.com/PicoQuant/PicoQuant-Time-Tagged-File-Format-Demos/blob/master/PTU/Python/Read_PTU.py.*
7. *https://github.com/Brinkslab/ptu_flim_tools/tree/master/ptu_flim_tools/third_party/readPTU_FLIM.*
